# The Tumor Suppressor SOCS1 Diminishes Tolerance to Oxidative Stress in Hepatocellular Carcinoma

**DOI:** 10.3390/cancers16020292

**Published:** 2024-01-10

**Authors:** Akhil Shukla, Md Gulam Musawwir Khan, Anny Armas Cayarga, Mozhdeh Namvarpour, Mohammad Mobarak H. Chowdhury, Dominique Levesque, Jean-François Lucier, François-Michel Boisvert, Sheela Ramanathan, Subburaj Ilangumaran

**Affiliations:** 1 Department of Immunology and Cell Biology, Faculty of Medicine and Health Sciences, Université de Sherbrooke, Sherbrooke, QC J1H 5N4, Canada; akhil.shukla@usherbrooke.ca (A.S.); gm.khan3937@gmail.com (M.G.M.K.); anny.armas.cayarga@usherbrooke.ca (A.A.C.); mozhdeh.namvarpour@usherbrooke.ca (M.N.); mohammad.mobarak.h.chowdhury@usherbrooke.ca (M.M.H.C.); dominique.levesque@usherbrooke.ca (D.L.); francois.michel.boisvert@usherbrooke.ca (F.-M.B.); sheela.ramanathan@usherbrooke.ca (S.R.); 2 Department of Biology, Université de Sherbrooke, Sherbrooke, QC J1K 2R1, Canada; jean-francois.lucier@usherbrooke.ca; 3 Centre de Recherche, Centre Hospitalier Universitaire de Sherbrooke, Sherbrooke, QC J1H 5N4, Canada

**Keywords:** hepatocellular carcinoma, SOCS1, tumor suppressor, oxidative stress, NRF2, cisplatin, tert-butyl hydroperoxide, mass spectrometry

## Abstract

**Simple Summary:**

SOCS1 functions as a tumor suppressor in liver cancer and in many other cancers, but the underlying mechanisms are not yet completely understood. Using mice lacking SOCS1 in hepatocytes, we have recently shown that SOCS1 deficiency increases the expression of NRF2. NRF2 is a key protein involved in regulating cellular oxidative stress. Whereas NRF2 is a tumor suppressor in normal cells, it can become an oncoprotein in cancer cells and confer resistance to oxidative stress. In this study, we examined the role of SOCS1 in liver cancer cells exposed to chemicals that induce oxidative stress and carried out a proteomic study. We show that SOCS1 attenuates NRF2 expression in HCC cells and reduces their ability to withstand oxidative stress by modulating diverse proteins involved in redox regulation. Hence, controlling the oxidative stress response is an important tumor suppression mechanism of SOCS1.

**Abstract:**

SOCS1 is a tumor suppressor in hepatocellular carcinoma (HCC). Recently, we showed that a loss of SOCS1 in hepatocytes promotes NRF2 activation. Here, we investigated how SOCS1 expression in HCC cells affected oxidative stress response and modulated the cellular proteome. Murine Hepa1-6 cells expressing SOCS1 (Hepa-SOCS1) or control vector (Hepa-Vector) were treated with cisplatin or tert-butyl hydroperoxide (*t*-BHP). The induction of NRF2 and its target genes, oxidative stress, lipid peroxidation, cell survival and cellular proteome profiles were evaluated. NRF2 induction was significantly reduced in Hepa-SOCS1 cells. The gene and protein expression of NRF2 targets were differentially induced in Hepa-Vector cells but markedly suppressed in Hepa-SOCS1 cells. Hepa-SOCS1 cells displayed an increased induction of reactive oxygen species but reduced lipid peroxidation. Nonetheless, Hepa-SOCS1 cells treated with cisplatin or *t*-BHP showed reduced survival. *GCLC*, poorly induced in Hepa-SOCS1 cells, showed a strong positive correlation with *NFE2L2* and an inverse correlation with *SOCS1* in the TCGA-LIHC transcriptomic data. A proteomic analysis of Hepa-Vector and Hepa-SOCS1 cells revealed that SOCS1 differentially modulated many proteins involved in diverse molecular pathways, including mitochondrial ROS generation and ROS detoxification, through peroxiredoxin and thioredoxin systems. Our findings indicate that maintaining sensitivity to oxidative stress is an important tumor suppression mechanism of SOCS1 in HCC.

## 1. Introduction

The suppressor of cytokine signaling 1 (SOCS1) is an inhibitor of the JAK-STAT signaling pathway induced by cytokines and is an indispensable regulator of inflammatory responses induced by Toll-like receptor and IFNγ signaling pathways [1,2,3,4,5,6,7,8]. SOCS1 is also implicated in regulating growth factor signaling via receptor tyrosine kinases [9,10,11,12,13,14]. The regulation of these pathways by SOCS1 can be mediated through the inhibition of tyrosine kinase activity and by binding to target signaling molecules and promoting their ubiquitination and subsequent degradation by proteasomes [15,16,17].

The notion that SOCS1 could be a tumor suppressor emerged from the finding that the *SOCS1* gene is frequently repressed in hepatocellular carcinoma (HCC) and in diverse other cancers such as gliomas, leukemias, lymphomas and colorectal, ovarian, breast and prostate cancers via the epigenetic methylation of CpG islands, as well as inactivation by mutations and microRNAs [18,19,20,21,22,23,24,25,26,27,28]. The propensity of SOCS1-deficient mice to develop radiation-induced leukemias and their increased susceptibility to the experimental induction of colorectal cancer and hepatocellular carcinoma confirm that SOCS1 is a *bona fide* tumor suppressor [29,30,31,32]. Mechanisms underlying the tumor suppressing activity of SOCS1 likely involve the regulation of multiple cancer-cell-signaling pathways. The first of these mechanisms involves the binding and inactivation of JAK kinases including the TEL-JAK oncoprotein, receptor tyrosine kinases such the hepatocyte growth factor receptor MET implicated in the progression of diverse cancers, and signaling proteins with oncogenic potential such VAV and ONCOVAV [13,15,33]. Secondly, SOCS1 can promote the activation of p53 and promote cellular senescence induced by oncogenes [34,35]. Thirdly, we have shown that SOCS1 is critical to regulate the tumor suppressor functions of the cyclin-dependent kinase inhibitor CDKN1A (p21) by preventing its cytosolic retention, which can render it oncogenic [31,32]. Among these potential mechanisms of SOCS1-mediated tumor suppression, only the attenuation of the pro-oncogenic capacity of CDKN1A has been genetically proven in our earlier study, wherein we demonstrated that the simultaneous ablation of the *Cdkn1a* gene attenuated HCC induction by the genotoxic hepatocarcinogen diethyl nitrosamine (DEN) in hepatocyte-specific SOCS1-deficient mice [32].

While characterizing the cellular signaling pathways deregulated by the loss of SOCS1 in hepatocytes, we observed an increased activation of the transcription factor ‘nuclear factor erythroid 2-related factor 2′ (NFE2L2, widely known as NRF2) in the livers of hepatocyte-specific SOCS1-deficient mice following exposure to DEN [31,32]. This was accompanied by a marked induction of many NRF2 target genes that regulate oxidative stress and maintain cellular redox homeostasis [36]. CDKN1A, which is upregulated by the loss of SOCS1, has been previously implicated in the activation of NRF2 [37,38]. CDKN1A has been shown to bind NRF2, dislodge its endogenous negative regulator KEAP1 and prevent the ubiquitination of NRF2 and its proteasomal degradation, which results in nuclear NRF2 accumulation and the transcription of its target genes. Despite an elevated induction of genes involved in reducing cellular oxidative stress, the livers of DEN-treated hepatocyte-specific SOCS1-deficient mice displayed increased staining of 4-hydoxynonenol (4-HNE), an indicator of lipid peroxidation [32,39], suggesting an elevated level of oxidative stress in SOCS1-deficient hepatocytes. This seemingly paradoxical scenario of increased lipid peroxidation despite an elevated expression of NRF2 target genes suggests that an increased expression of antioxidant response genes in SOCS1-deficient hepatocytes might enable them to better tolerate oxidative stress, which in turn could contribute to cancer cell growth and tumor progression.

The aim of the current study is to fully understand the functions of SOCS1 in modulating oxidative stress response in HCC cells. The basal expression of SOCS1 is generally low in many immune cells and parenchymal cells. SOCS1 is induced by cytokines and growth factors as a negative feedback inhibitor, as well as by myriad of other signals, including bacterial lipopolysaccharide and genotoxic stressors [7,31,32,34,40]. Therefore, to mimic the condition of SOCS1 expression, we stably expressed SOCS1 in murine and human HCC cell lines and treated them with chemical agents to induce oxidative stress and studied NRF2 activation and carried out a proteomic analysis. Our findings show that SOCS1 expression in HCC cells is associated with reduced NRF2 activation, attenuates the induction of NRF2 target genes and increases their susceptibility to cell death induced by oxidative stress. A proteomic analysis of SOCS1 expressing cells exposed to oxidative stress revealed that SOCS1 regulates multiple cellular signaling pathways, including those involved in chemical carcinogenesis and oxidative stress response, within and outside the NRF2-mediated antioxidant pathway.

## 2. Materials and Methods

### 2.1. Cell Lines and Treatment

Hepa1-6 (Hepa) murine hepatoma (ATCC: CRL1830) and Hep3B human HCC (HB-8064) cell lines, authenticated by short-tandem repeat profiling, were purchased from ATCC. The two lines, Hepa1-6 and Hep3B, do not appear in the misidentified cell line list maintained by the International Cell Line Authentication Committee (ICLAC; https://iclac.org/databases/cross-contaminations/; version 12; accessed 1 June 2023). The establishment of stable lines of Hepa and Hep3B cells expressing SOCS1 (Hepa-SOCS1; Hep3B-SOCS1) or the control vector (Hepa-Vector; Hep3B-Vector) were previously described [12,13]. Hepa cells were transduced with lentiviral vector pWPT carrying N-terminal HA-tagged *SOCS1* gene or empty vector, and stable lines were established after sorting for the human CD8 marker in the vector [12]. Hep3B cells were transfected with pCDNA3.1 vector carrying N-terminal Myc tagged *SOCS1* gene or control vector and stable lines were established by G418 selection [13]. Both cell lines were grown in Dulbecco’s modified Eagle’s medium (DMEM) containing 10% fetal bovine serum (FBS). Cells were treated with the indicated concentrations of cisplatin (*cis*-diamino-dichloroplatinum; *cis*-Pt; Sigma-Aldrich (St. Louis, MO, USA), Cat# C2210000) or *tert*-butyl hydroperoxide (*t*-BHP; Sigma-Aldrich, Cat# 458139) to induce oxidative stress for the indicated periods of time.

### 2.2. Western Blot

Cell lysates from control and treated cells were lysed in 1x SDS-PAGE sample buffer and proteins separated on SDS-PAGE gels were transferred to polyvinylidene difluoride membranes and probed with the primary antibodies listed in Supplementary Table S1. Secondary antibodies and enhanced chemiluminescence reagents were purchased from GE Healthcare Life Sciences (Pittsburg, PA, USA). Western blot images were captured by the VersaDOC 5000 imaging system (Bio-Rad, Hercules, CA, USA).

### 2.3. Gene Expression

Total RNA from control and cisplatin- or *t*-BHP-treated Hepa-vector, Hepa-SOCS1, Hep3B-vector and Hep3B-SOCS1 cells were collected in RNAlater^®^ (ThermoFisher Scientific, Waltham, MA, USA) and stored frozen. RNA was isolated using RNeasy^®^ kit (Qiagen, Hilden, Germany) and cDNA synthesized using QuantiTect^®^ reverse transcription kit (Qiagen). Real-time quantitative PCR analysis was carried out on the MyQi5^®^ cycler (Bio-Rad) using primers with 90–100% efficiency and a single melting curve that are listed in Supplementary Table S2. Gene expression in the treated cells was normalized to the reference gene *Rplp*0 and mRNA fold-induction was calculated relative to the expression in respective untreated cell lines.

### 2.4. Immunofluorescence (IF) Microscopy

Cells were grown in glass coverslips and treated with cisplatin or *t*-BHP. At the indicated time points, cells were washed in PBS and fixed overnight in cold PBS containing 4% paraformaldehyde (PFA). The cover slips were washed in PBS, blocked with PBS containing 5% BSA and 0.3% Triton X-100, and incubated overnight at 4 degrees with primary antibodies (Supplementary Table S1). After thorough washing in PBS containing 0.1% Tween-20 (PBST), the coverslips were incubated with secondary antibodies conjugated to fluorochrome in a dark chamber for 60 min at room temperature. After washing with PBST, the coverslips were immersed in Hoechst 33342 (Thermo Fisher Scientific, Cat# H3570) for 5–10 min, washed in PBS and mounted on glass slides using Dako Fluorescence Mounting Medium (Agilent Technologies, Santa Clara, CA, USA, S3023). The images were captured using the Axioskop 2 microscope (Carl Zeiss, Inc., Thornwood, NY, USA).

### 2.5. Oxidative Stress

Cells were grown in glass coverslips and treated with cisplatin or *t*-BHP and were fixed in 4% PFA. Oxidative stress was evaluated using the CellROX Green and CellROX Deep Red fluorescent ROS indicators (Molecular Probes; ThermoFisher Scientific; Cat #C10444 and C10422, respectively) following the manufacturer’s instructions. Lipid peroxidation was evaluated by the IF staining of 4-hydroxynonenol (4-HNE).

### 2.6. Cell Viability

Cell survival in cisplatin- or *t*-BHP-treated cells was measured using the WST-8 (water-soluble Tetrazolium-8: 2-(2-methoxy-4-nitrophenyl)-3-(4-nitrophenyl)-5-(2,4-disulfophenyl)-2*H*-tetrazolium) assay kit (CCK-8; Dojindo Molecular Technologies, #CK04) following the manufacturer’s instructions. Briefly, 3000 cells were seeded in 96-well plates in 100 μL medium in the presence or absence of cisplatin or *t*-BHP. After the indicated periods of incubation, 100 μL WST-8 reagent was added to each well and incubated for 2 h. Color development, indicative of cell number and viability, was measured at 440 nm wavelength using a SPECTROstar^Nano^ (BMG Labtech, Ortenberg, Germany) spectrometer.

### 2.7. Statistical Analysis

Data analysis and graphics representation were carried out using GraphPad Prism Version 10.0.3 (San Diego, CA, USA) and represented as mean ± standard error of mean (SEM). Statistical significance was calculated by ANOVA with Tukey’s multiple comparison test and *p* values are represented by asterisks: * <0.05, ** <0.01, *** <0.001, **** <0.001.

### 2.8. TCGA-LIHC Dataset and Analyses

The Cancer Gene Atlas-liver HCC (TCGA-LIHC) dataset (PanCancer Atlas) containing transcriptomic data on 366 cases was accessed via the cBioPortal (https://www.cbioportal.org; accessed on 17 October 2023) to evaluate the correlation between the transcript levels of *SOCS1*, *NFE2L2* and NRF2 target genes.

### 2.9. Mass Spectrometry

#### 2.9.1. Protein Preparation and Protease Digestion

For the extraction of total protein, cells grown in 100 mm Petri dishes and treated with cisplatin or *t*-BHP for 3 h were sedimented, washed in PBS and resuspended in 500 µL of lysis buffer (8 M Urea, 1 M NH_4_HCO_3_ and 10 mM HEPES-KOH pH7.5). Cell lysates were transferred to 1.5 mL low protein binding tubes (Axygen, Union City, CA, USA, cat #MCT-150-C) and sonicated on ice (12 cycles, 20–25% intensity, 5 s PULSE/5 sec OFF) and centrifuged at 16,000× *g* for 10 min at 4 °C. Supernatants were transferred to fresh low protein binding tubes and proteins were quantified using DC Protein Assay Kit (Bio-Rad, #5000113, #5000114, #5000115) following the manufacturer’s instructions. Then, 50 µg of protein was transferred to new low protein binding tubes, volumes adjusted to 50 µL with urea solution (8 M Urea, 10 mM HEPES, pH 8) and 1 µL of 255 mM dithiothreitol was added to achieve a final concentration of 5 mM. The tubes were vortexed and boiled at 95 °C for 2 min and allowed to cool at room temperature for 30 min. After adding 1.5 µL of photosensitive 262.5 mM chloroacetamide to achieve a 7.5 mM final concentration, the samples were vortexed and incubated in the dark at room temperature for 20 min before adding 150 µL of 50 mM NH_4_HCO_3_ to bring the urea concentration down to 2 M. Proteins were digested by adding 1 µg of Pierce^TM^ Trypsin Protease (Thermo Scientific, cat# 90058) followed by overnight incubation at 30 °C. Proteolysis was stopped by adding 0.508 µL of 100% Trifluoroacetic acid (TFA) and the digested peptides were cleaned using Pierce^TM^ C18 tips (Thermo Scientific, cat# 87784). The solvents were then removed using Vacufuge Plus centrifuge concentrator (Eppendorf, Hamburg, Germany) and the peptides diluted in 1% Formic Acid (FA) were quantified using NanoDrop 2000/2000c spectrophotometer (Thermo Fisher scientific).

#### 2.9.2. Liquid Chromatography-Tandem Mass Spectrometry (LC-MS/MS)

Concentrated peptides (250 ng) were separated on a nanoHPLC system (nanoElute, Bruker Daltonics, Billerica, MA, USA). The samples were loaded onto an Acclaim PepMap100 C18 Trap Column (0.3 mm id × 5 mm, Dionex Corporation, Sunnyvale, CA, USA) at 4 µL/min consistent flow and peptides were eluted onto a PepMap C18 analytical nanocolumn (1.9 µm beads size, 75 µm × 25 cm, PepSep, Marslev, Denmark) heated at 50 °C. Peptides were eluted with solvent B (100% ACN and 0.1% FA) in a 5–37% linear gradient with a flowrate of 400 nL/min for ~2 h. The HPLC system was coupled to a TimsTOF Pro ion mobility mass spectrometer containing Captive Spray nano electrospray source (Bruker Daltonics). Data acquisition was undertaken using diaPASEF mode. For each individual Trapped Ion Mobility Spectrometry (TIMS) measurement in diaPASEF mode, a single mobility window consisting of 27 mass steps (with *m*/*z* ranging from 114 to 1414 and a mass width of 50 Da) was employed per cycle, with a 1.27 s duty cycle. This process involves scanning the diagonal line in the *m*/*z*-ion mobility plane for +2 and +3 charged peptides.

#### 2.9.3. Protein Identification

Peptide mass spectra were analyzed using the DIA-NN [41], an open-source software suite for DIA/SWATH data processing (https://github.com/vdemichev/DiaNN, version 1.8.1; accessed 23 August 2023), installed in an Apptainer container (https://apptainer.org/; accessed 23 August 2023) using the docker image provided on the docker hub [42]. Analysis was performed using default parameters except for these options: 2 missed cleavages were allowed; trypsin digestion was performed for K/R; and with protein N-term methionine excision as variable modification for in silico digest.

The *Mus musculus* reference proteome UP000000589 was downloaded from the Uniprot website (https://www.uniprot.org/proteomes/UP000000589; accessed 25 August 2023). The reference proteome contained a total of 63,367 proteins. For the FASTA search, DIA-NN was instructed to perform an in silico digest of the sequence database. A mass tolerance accuracy of MS1 and MS2 of 20 ppm was used for precursor and fragment ions, respectively. The minimum and maximum were set for peptide length (7–30 amino acids), and the precursor charge (1–5), precursor *m*/*z* (100–1700) and fragmentation *m*/*z* (100–1500) for in silico library generation or library-free search. For the reanalysis, MBR (match between run) was enabled and smart profiling was chosen when creating a spectral library from DIA data. Carboxyamidomethylation (unimod4) and oxidation (M) (unimod35) were set as fixed modifications and N-terminal protein acetylation was set as a variable modification.

#### 2.9.4. Proteomic Data Visualization

Microsoft Excel (Office 365 Version 2310 Build 16.0.16924.20054) was used to sort significantly modulated proteins with cut-off values ≥ 0.5 (fold change) and ≥1.3 (-log10 *p*-Value). Curated datasets and comparisons between groups are provided in Supplementary Table S3. GraphPad Prism version 10.0.3 (GraphPad, Boston, MA, USA) was used to generate volcano plots and pie charts. Venn diagrams were generated using the jvenn (https://jvenn.toulouse.inrae.fr/app/index.html; accessed on 10 October 2023) online tool [43]. The SRplot server (http://www.bioinformatics.com.cn/srplot; accessed on 27 October 2023) was used for pathway and Gene Ontology (GO) analyses and to generate pathway enrichment plots, heatmaps and cnetplot.

## 3. Results

### 3.1. SOCS1 Inhibits NRF2 Induction through Oxidative Stress

**Figure 1:**
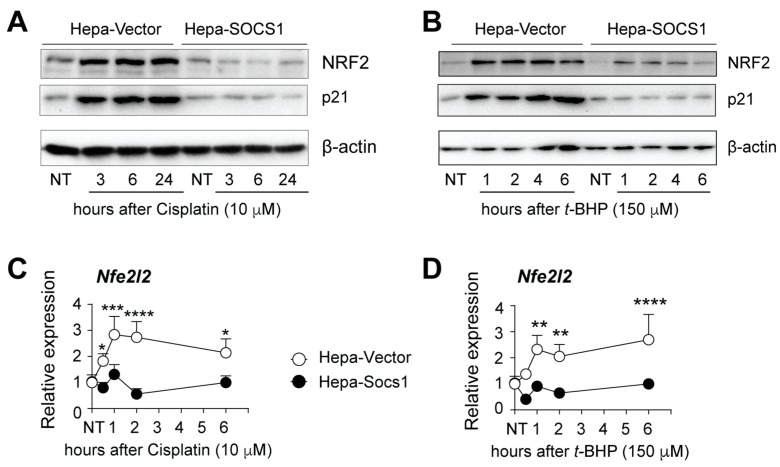
SOCS1 diminishes NRF2 expression induced by oxidative stress. Hepa 1-6 cells expressing control vector (Hepa-Vector) or SOCS1 (Hepa-SOCS1) were treated with cisplatin (10 μM; (**A**)) or *t*-BHP (150 μM; (**B**)) and evaluated for the expression of p21 (CDKN1A) and NRF2 (NFE2L2) proteins using Western blot. β-actin served as a loading control. Representative data from two independent experiments are shown. Hepa-vector and Hepa-SOCS1 cells treated with cisplatin (**C**) or *t*-BHP (**D**) for the indicated periods of time and the expression of *Nfe2l2* gene were evaluated by RT-qPCR. Cumulative data from four separate experiments are shown as mean ± standard error of mean (SE). ANOVA with Tukey’s multiple comparison test. *p* values: * <0.05, ** <0.01, *** <0.001, **** <0.001. The uncropped blots are provided at the end of the Supplementary Figures.

To study the impact of SOCS1 expression on antioxidant responses in HCC cells, we treated the murine Hepa1-6 (Hepa) hepatoma cell line stably expressing SOCS1 (Hepa-SOCS1) or the empty vector (Hepa-Vector) with cisplatin or *t*-BHP, which are known to induce oxidative stress [44,45,46,47,48,49,50,51,52,53]. Cisplatin increases mitochondrial ROS production, whereas *t*-BHP depletes antioxidant defense systems and also reduces mitochondrial membrane potential. Exposure to cisplatin markedly induced NRF2 protein expression in Hepa-Vector cells within 3 h after treatment, and this continued to remain elevated for up to 24 h (Figure 1A). Similarly, *t*-BHP treatment upregulated NRF2 protein expression within 1 h, which lasted for at least 6 h in Hepa-Vector cells (Figure 1B). On the other hand, NRF2 upregulation by both cisplatin and *t*-BHP was markedly attenuated in Hepa-SOCS1 cells (Figure 1A,B). The increase in NRF2 expression in Hepa-Vector cells following cisplatin or *t*-BHP treatment coincided with a marked upregulation of p21 protein expression, which was also abolished by stable SOCS1 expression (Figure 1A,B). The SOCS1-mediated attenuation of NRF2 and p21 protein expression also occurred in human Hep3B hepatoma cells expressing SOCS1 (Hep3B-SOCS1) exposed to *t*-BHP (Supplementary Figure S1A). Consistent with the fact that NRF2 induces its own transcription [54,55], exposure to cisplatin or *t*-BHP caused a rapid two- to four-fold increase in the expression of the *Nfe2l2* gene within 1 h after drug treatment, which remained elevated for up to 6 h in Hepa-vector cells, whereas Hepa-SOCS1 cells did not show an appreciable induction of the *Nfe2l2* gene (Figure 1C,D). These data indicate that SOCS1 is a key regulator of NRF2 protein expression and its transcriptional activation in hepatoma cells exposed to oxidative stress.

### 3.2. SOCS1 Inhibits the Induction of NRF2 Target Genes and Proteins

**Figure 2:**
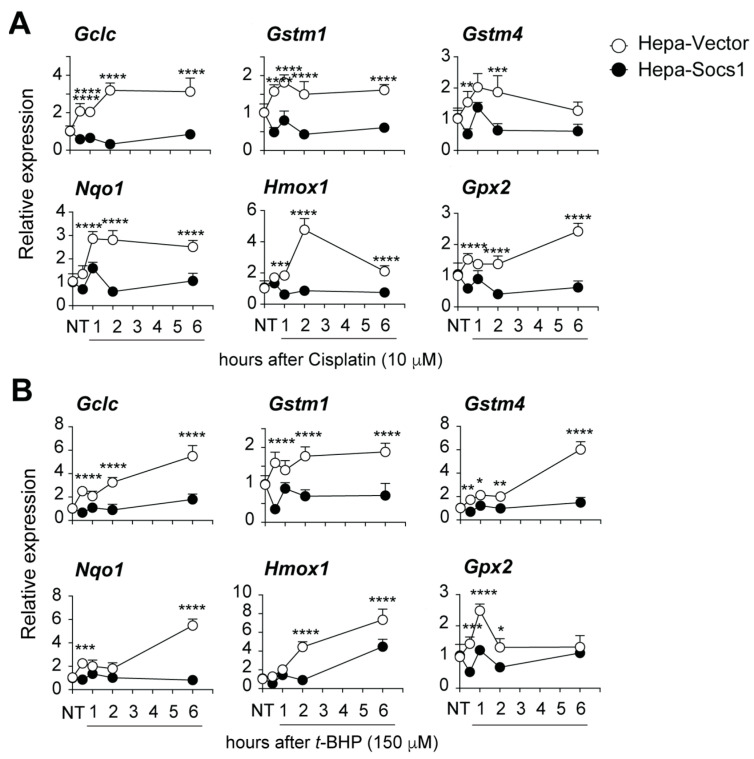
SOCS1 inhibits the induction of NRF2 target genes following oxidative stress. Hepa-Vector and Hepa-SOCS1 cells treated with cisplatin (10 μM; (**A**)) or *t*-BHP (150 μM; (**B**)) for the indicated periods of time and the expression of the indicated NRF2 target genes were evaluated by RT-qPCR. Cumulative data from four separate experiments are shown as mean ± SE. ANOVA with Tukey’s multiple comparison test. *p* values: * <0.05, ** <0.01, *** <0.001, **** <0.001.

Next, we compared the induction of NRF2 target genes involved in cellular oxidative stress response in Hepa-Vector and Hepa-SOCS1 cells at different time points following exposure to cisplatin or *t*-BHP. The NRF2 target genes *Gclc*, *Gstm1*, *Gstm4* and *Nqo1* were rapidly induced in cisplatin-treated Hepa-Vector cells, attaining the peak expression within 1 h, and remained elevated for up to 6 h, except *Gstm4*, which declined (Figure 2A). *Hmox1* and *Gpx2* genes were induced by cisplatin with delayed kinetics in Hepa-Vector cells, reaching peak levels after 2 h and 3 h, respectively (Figure 2A). On the other hand, *t*-BHP induced *Gclc*, *Gstm4*, *Nqo1* and *Hmox1* genes more strongly than cisplatin, with expression levels continuing to rise for up to 6 h, although the induction of *Gpx2* declined after 1 h (Figure 2B). The expression of all these NRF2 target genes was significantly attenuated in Hepa-SOCS1 cells, without showing any appreciable upregulation except *Hmox1*, which increased in Hepa-SOCS1 cells after 6 h exposure to *t*-BHP (Figure 2A,B).

**Figure 3:**
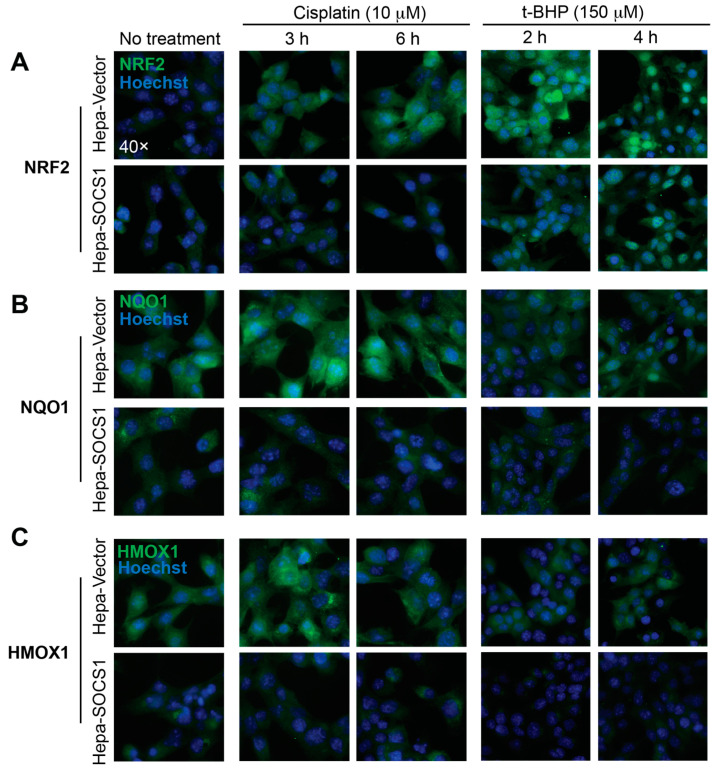
SOCS1 diminishes the NRF2 target protein expression induced by oxidative stress. Hepa-Vector and Hepa-SOCS1 cells grown on glass cover slips were treated with cisplatin (10 μM) or *t*-BHP (150 μM) for the indicated periods of time. The expressions of the NRF2 (**A**), NQO1 (**B**) and HMOX1 (**C**) proteins were evaluated by immunofluorescence microscopy. Images of all control and treated Hepa-Vector and Hepa-SOCS1 cells for each indicated antibody were captured at same settings. Representative data from two experiments are shown. Secondary antibody staining of Hepa-Vector cells without the first antibody did not give any detectable signal at the image capture settings used.

The evaluation of the expression of NRF2 by immunofluorescence revealed a more potent induction in *t*-BHP-treated Hepa-Vector cells than in cisplatin-treated cells (Figure 3A), consistent with the increase in *Nfe2l2* transcript levels (Figure 1C,D). Notably, NRF2 localized to the nucleus at 6 h and 4 h after cisplatin or *t*-BHP treatment, respectively (Figure 3A). NRF2 protein expression was barely detectable in cisplatin-treated Hepa-SOCS1 cells, which showed a few cells with nuclear NRF2 after *t*-BHP treatment for 6 h (Figure 3A). Compared to NRF2, NQO1 and HMOX1 (HO-1) proteins showed higher levels of basal expression and were strongly induced by cisplatin in Hepa-Vector cells (Figure 3B,C). The protein levels of NQO1 and HMOX1 were not appreciably increased at 4 h after *t*-BHP treatment, consistent with the observation that *Nqo1* and *Hmox1* gene induction in Hepa-Vector cells peaked only at 6 h (Figure 2B and Figure 3B,C). The protein expressions of NRF2, NQO1 and HMOX1 at the basal level and in cisplatin- or t-BHP-treated Hepa-SOCS1 cells remained low (Figure 3A–C). On the other hand, both agents induced SOCS3 protein expression to a comparable extent in Hepa-Vector and Hepa-SOCS1 cells (Supplementary Figure S2), indicating that stable SOCS1 expression specifically impacts proteins involved in oxidative stress response.

### 3.3. SOCS1 Increases Oxidative Stress and Reduces Cell Survival

**Figure 4:**
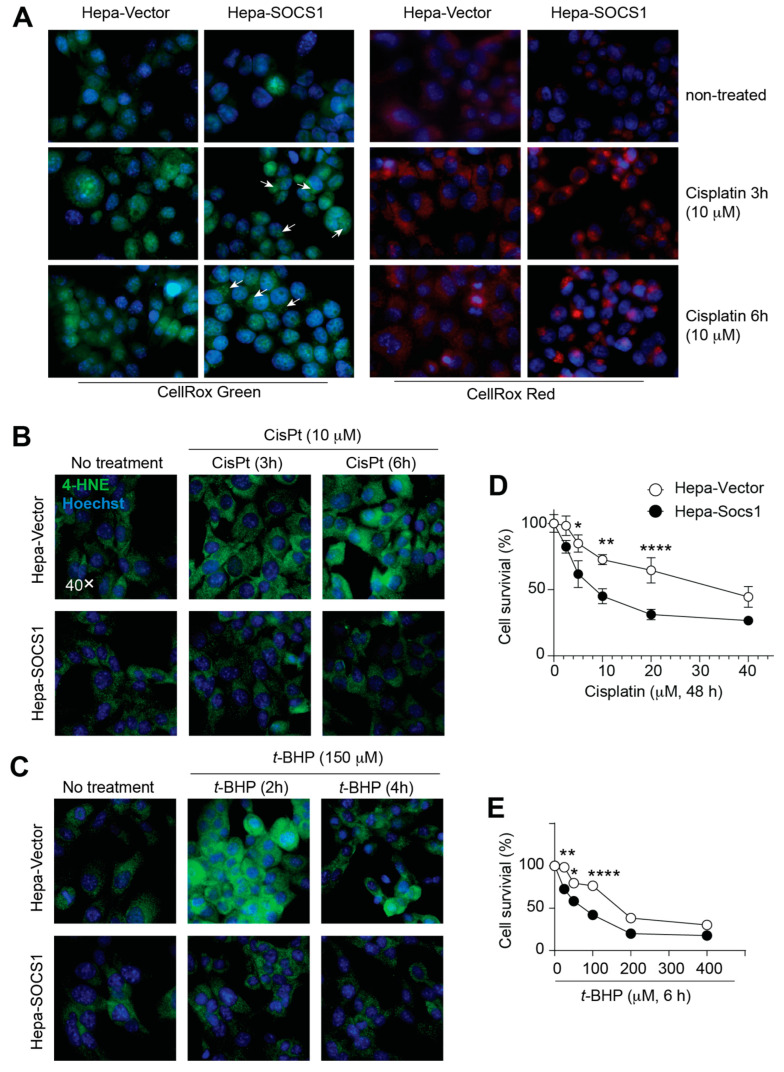
SOCS1 increases oxidative stress and reduces cell survival without increasing lipid peroxidation. (**A**,**B**) Hepa-Vector and Hepa-SOCS1 cells grown on glass covers were treated with cisplatin (10 μM) for the indicated periods of time. Oxidative stress was evaluated by CellRox staining. Mitochondrial CellRox Green staining is indicated by arrows. (**B**,**C**) Hepa-Vector and Hepa-SOCS1 cells were treated with cisplatin (10 μM) or *t*-BHP (150 μM) for the indicated periods of time. Lipid peroxidation was evaluated by 4-HNE immunostaining with DAPI staining of nuclei. (**D**,**E**) Hepa-Vector and Hepa-SOCS1 were treated with the indicated concentrations of cisplatin for 48 h or *t*-BHP for 6 h and cell survival was evaluated by the WST-8 assay. For (**A**–**C**), representative data from two experiments are shown. For (**D**,**E**), pooled data from three experiments are shown. Mean ± SE; Two-way ANOVA with Tukey’s multiple comparison test; * *p* < 0.05, ** *p* < 0.01, **** *p* < 0.0001.

To evaluate oxidative stress following exposure to cisplatin, we used CellRox Green and CellRox Red stains. Upon oxidation, CellRox Green binds to DNA and thus its signal is localized mainly in the nucleus and mitochondria, whereas the CellRox Red signal is localized to the cytoplasm. Hepa-Vector cells displayed discernibly more basal CellRox Green staining than Hepa-SOCS1 cells (Figure 4A, left panels). Cisplatin caused a marked increase in CellRox Green staining that was markedly elevated in Hepa-SOCS1 cells compared to Hepa-Vector cells. Moreover, Hepa-SOCS1 cells displayed more distinct mitochondrial staining (Figure 4A, arrows) and intense nuclear staining after cisplatin treatment for 6 h. CellRox Red revealed diffuse cytoplasmic staining in untreated Hepa-Vector cells that was markedly elevated following cisplatin treatment (Figure 4A, right panels). Hepa-SOCS1 cells showed reduced basal CellRox Red staining and displayed an intense, localized staining pattern. These observations indicated that SOCS1 expression increases cisplatin-induced oxidative stress, consistent with a reduced expression of antioxidant proteins.

The evaluation of membrane lipid peroxidation resulting from oxidative stress by the immunostaining of 4-hydroxynonenal (4-HNE) [39] revealed that Hepa-Vector cells showed an appreciable increase in 4-HNE staining at 3 h after exposure to cisplatin that increased further until 6 h, whereas *t*-BHP caused a rapid increase in 4-HNE staining within 2 h of treatment, which was reduced by 4 h (Figure 4B,C). Intriguingly, Hepa-SOCS1 cells did not show any discernible increase in 4-HNE levels following exposure to cisplatin or *t*-BHP despite the increased generation of ROS (Figure 4A–C). Nonetheless, Hepa-SOCS1 cells showed significantly reduced cell survival following cisplatin or *t*-BHP treatment (Figure 4D,E). Human Hep3B cells expressing SOCS1 also showed reduced survival (Supplementary Figure S1B). These data suggest that SOCS1 inhibits the ability of hepatoma cells to withstand elevated levels of oxidative stress.

### 3.4. SOCS1 Expression in HCC Negatively Correlates with NFE2L2 and GCLC Expression

**Figure 5:**
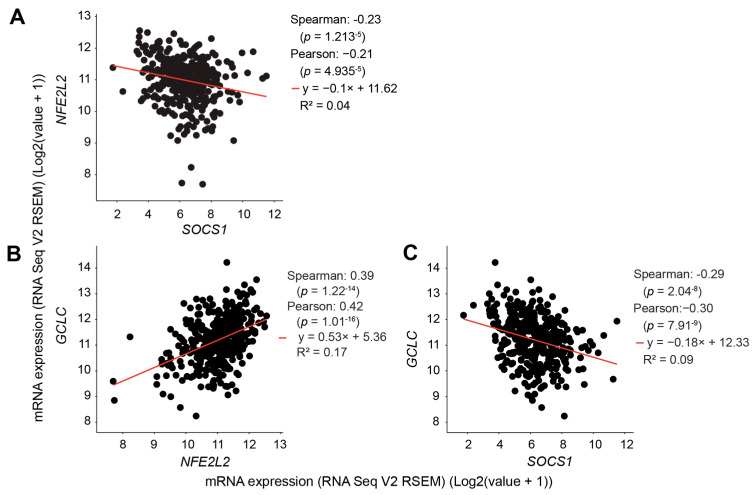
High *SOCS1* gene expression in HCC correlates with low *NFE2L2* and *GCLC* gene expression in the TCGA-LIHC transcriptomic data. Correlations between the expression of *SOCS1* and *NFE2L2* (**A**), *NFE2L2* and *GCLC* (**B**) and *SOCS1* and *GCLC* (**C**) genes in the TCGA-LIHC transcriptomic data were analyzed using the cBioportal platform. Correlation coefficient, significance values and slope are indicated.

Among the NRF2 target genes induced by oxidative stress and attenuated by SOCS1, some are involved in redox homeostasis mediated by glutathione [56]. GCLC (glutamate cysteine ligase catalytic subunit) is part of the rate-limiting enzyme in glutathione biosynthesis [57]. *GCLC* induction by cisplatin or *t*-BHP is markedly attenuated in Hepa-SOCS1 cells compared to Hepa-Vector cells (Figure 2). Since low *SOCS1* gene expression correlates with poor prognosis in the TCGA-liver hepatocellular carcinoma (LIHC) dataset [32], and high GCLC in HCC tissues correlates with poor prognosis [58], we evaluated the relationship between *SOCS1, NFE2L2* (NRF2) and *GCLC* gene expression in the TCGA-LIHC dataset. Increased *SOCS1* gene expression showed a significantly negative correlation with *NFE2L2* gene expression, which showed a significant positive correlation with *GCLC* transcript levels (Figure 5A,B). Notably, *SOCS1* gene expression showed a highly significant inverse correlation with *GCLC* gene expression (Figure 5C). Intriguingly, *NFE2L2* gene expression did not show any significant correlation with several known NRF2 target genes such as *GSTM1*, *GSTM4*, *GPX4*, *HMOX1* and *NQO1*, while *SOCS1* even showed a significant positive correlation with *HOMX1* and *NQO1* within the TCGA-LIHC transcriptomic data (Supplementary Figure S3). These observations suggest variable NRF2 target gene expression in different cell types, and the unknown cell purity of TCGA specimens used for RNAseq may influence the correlation analysis. Nonetheless, our findings support the notion that the SOCS1-mediated downregulation of *NFE2L2* expression likely impacts at least a subset of NRF2 target genes such as *GCLC* in HCC.

### 3.5. SOCS1 Expression Profoundly Modulates the Proteome of Hepatoma Cells

**Figure 6:**
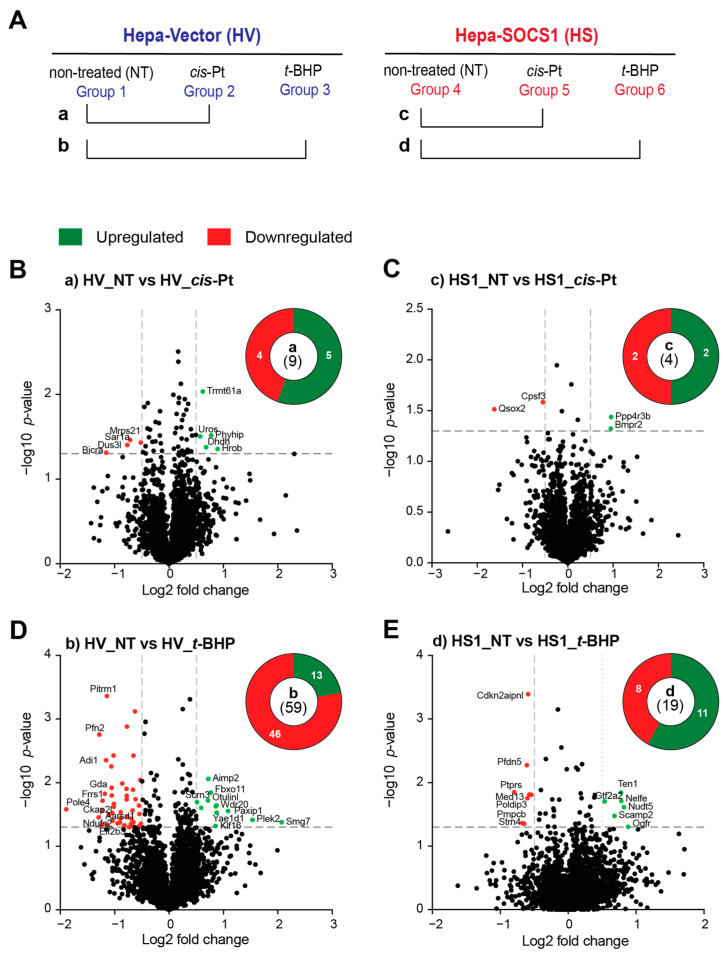
Impact of oxidative stress on the total proteome of Hepa cells. (**A**) Vector and Hepa-SOCS1 cells grown in 100 mm Petri dishes were treated with cisplatin (10 μM) or *t*-BHP (150 μM) for 3 h or left untreated. Proteins extracted from three different cultures (biological replicates) were subjected to shotgun proteomic analysis, and their proteomic profiles were compared as indicated (**a**, **b**, **c**, **d**). (**B**–**E**) Volcano plots showing significantly modulated proteins in Hepa-Vector cells treated with cisplatin (**B**) or t-BHP (**C**) compared to non-treated cells, and in Hepa-SOCS1 cells treated with treated with cisplatin (**D**) or t-BHP (**E**) compared to non-treated cells. Upregulated and downregulated proteins are indicated in green and red color dots, respectively. Pie charts indicate the total number of modulated proteins within the parenthesis in the center and the numbers of upregulated and downmodulated proteins with the green and red segments, respectively.

To understand the mechanisms of SOCS1-dependent regulation of elevated oxidative stress in hepatoma cells, we carried out a mass spectrometry analysis of the total proteome of the Hepa-vector and Hepa-SOCS1 cells after exposure to cisplatin or *t*-BHP for 3 h. The proteomic data were first analyzed to compare cisplatin- or *t*-BHP-treated cells to their respective untreated controls (Figure 6A). Cisplatin treatment caused minimal changes in the proteome of Hepa-Vector and Hepa-SOCS1 cells, with only a very few significantly downregulated and upregulated proteins (Figure 6B,C). Exposure to *t*-BHP caused more changes, relatively, in the proteome of both cell lines (Figure 6D,E). Whereas the number of downregulated proteins were more numerous than the upregulated proteins in Hepa-Vector cells, they were fewer and comparable in Hepa-SOCS1 cells (Figure 6D,E).

**Figure 7:**
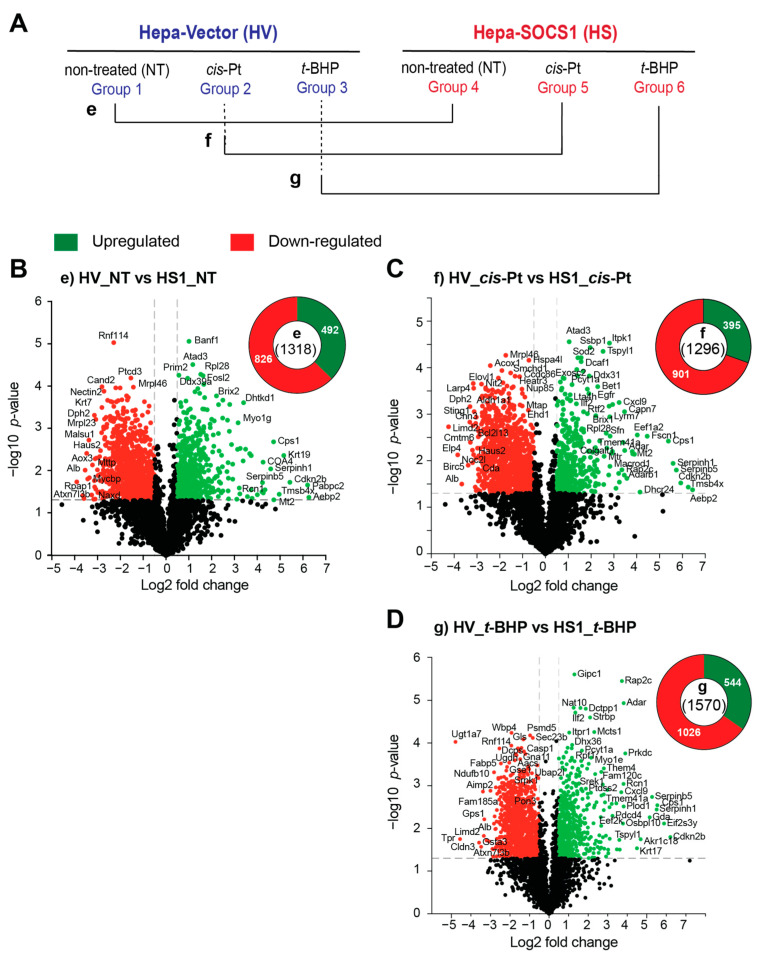
SOCS1 causes profound changes to the proteome of hepatoma cells. (**A**) The proteomic profiles of untreated and cisplatin-treated or *t*-BHP-treated Hepa-SOCS1 cells were compared to those of Hepa-Vector cells, as indicated (**e**, **f**, **g**). (**B**–**D**) Volcano plots showing significantly modulated proteins in untreated (**B**), cisplatin treated (**C**) and *t*-BHP treated (**D**) Hepa-SOCS1 cells compared to respective Hepa-Vector cells. Upregulated and downregulated proteins are indicated in green and red color dots, respectively. Pie charts indicate the total number of modulated proteins within parenthesis in the center and the numbers of upregulated and downmodulated proteins with the green and red segments, respectively.

Next, we compared the proteome of Hepa-Vector and Hepa-SOCS1 cells without any treatment and after exposure to cisplatin or *t*-BHP, as depicted in Figure 7A. Surprisingly, hundreds of proteins were found to be significantly downregulated (826 proteins) or upregulated (492 proteins) in untreated Hepa-SOCS1 cells compared to Hepa-Vector controls (Figure 7B). Exposure to cisplatin or *t*-BHP further increased the number of downregulated (901, 1026 proteins, respectively) or upregulated (395, 544 proteins, respectively) proteins in Hepa-SOCS1 cells compared to Hepa-Vector controls (Figure 7C,D). Even though the downregulated proteins were more numerous than upregulated proteins in Hepa-SOCS1 cells compared to Hepa-Vector cells in all cases (untreated, cisplatin-treated, *t*-BHP-treated), the upregulated proteins still represented about one third of the significantly modulated proteins. These observations indicated that SOCS1 causes profound changes to the proteome of hepatoma cells, which is further accentuated under conditions of oxidative stress.

### 3.6. Oxidative Stress Response Is a Major Biological Pathway Modulated by SOCS1

**Figure 8:**
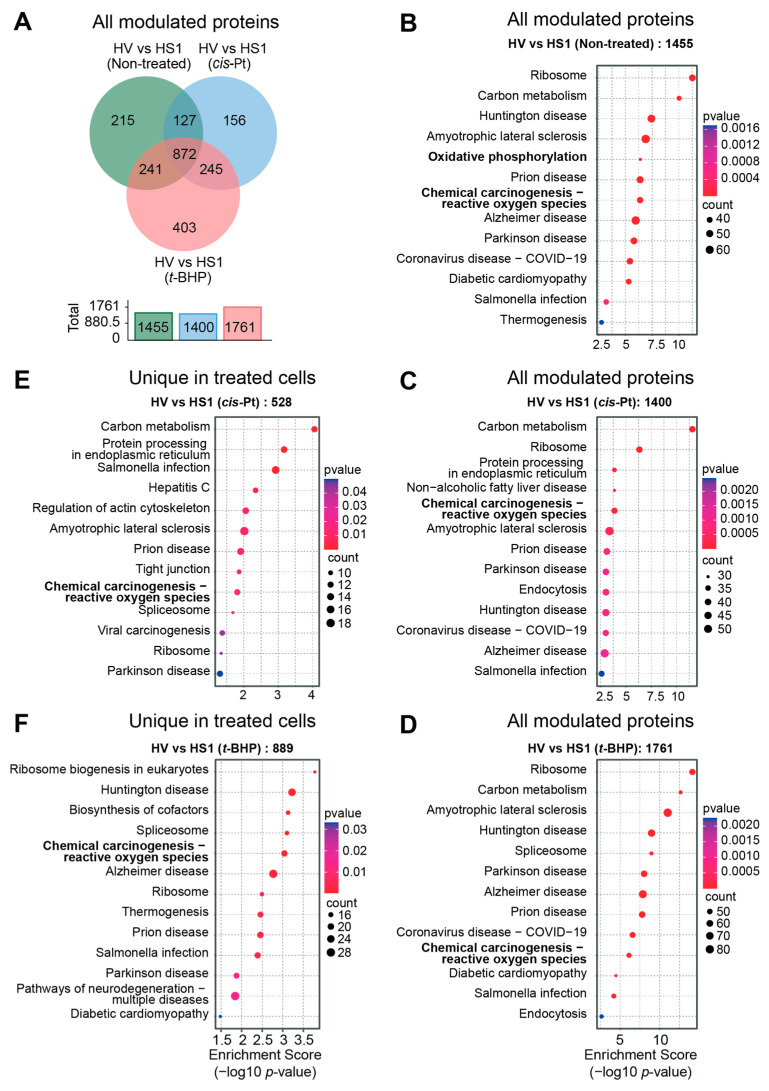
Pathway analysis of proteins modulated by SOCS1. (**A**) Venn diagram showing the total numbers of proteins modulated in untreated, cisplatin or *t*-BHP treated Hepa-SOCS1 cells compared to respective Hepa-Vector cells. Total number of modulated proteins are indicted in the histogram. (**B**–**D**) Pathway analysis of all proteins differentially expressed in untreated (**B**), cisplatin-treated (**C**) or *t*-BHP-treated (**D**) Hepa-SOCS1 cells. (**E**,**F**) Pathway analysis of proteins uniquely modulated in cisplatin-treated (**E**) or *t*-BHP treated (**F**) Hepa-SOCS1 cells.

A comparison of the proteins modulated in Hepa-SOCS1 cells compared to Hepa-Vector cells revealed that more than half of the proteins modulated by SOCS1 are shared at the basal level (872/1455) and after exposure to cisplatin (872/1400) or *t*-BHP (872/1761) (Figure 8A). In addition, a significant number of proteins modulated by SOCS1 were shared between untreated and cisplatin-treated (127) or *t*-BHP-treated (241) cells, and cisplatin-treated and *t*-BHP-treated (245) cells. SOCS1 also modulated a unique set of proteins in untreated (215), cisplatin-treated (156) and *t*-BHP-treated (403) cells, indicating that SOCS1 profoundly modulates the hepatocyte proteome before and after exposure to oxidative stress.

A pathway analysis of all the proteins modulated by SOCS1 in Hepa cells revealed that ribosomal proteins and those involved in ‘carbon metabolism’ (a KEGG reference pathway that includes central carbohydrate metabolism pathways—glycolysis, tricarboxylic acid cycle and pentose-phosphate pathway) represent the predominant and most significantly modulated pathway proteins in untreated and cisplatin- or *t*-BHP-treated cells (Figure 8B–D). Notably, proteins involved in the ‘chemical carcinogenesis–reactive oxygen species’ pathway are significantly modulated by SOCS1 in untreated cells as well as in cisplatin- or *t*-BHP-treated Hepa-SOCS1 cells. An analysis of proteins modulated by SOCS1 only in cisplatin- or *t*-BHP-treated cells (unique in treated cells) also showed a significant enrichment of the ‘chemical carcinogenesis–reactive oxygen species’ pathway proteins (Figure 8E,F). Moreover, proteins modulated by SOCS1 only in cisplatin-treated cells showed an enrichment of the ‘carbon metabolism’ pathway (Figure 8E). These findings indicate that SOCS1 is a key regulator of cellular redox homeostasis in hepatocytes at the basal level and additionally upon exposure to oxidative stress.

### 3.7. SOCS1 Downregulates the Oxidative Stress Response Pathway

**Figure 9:**
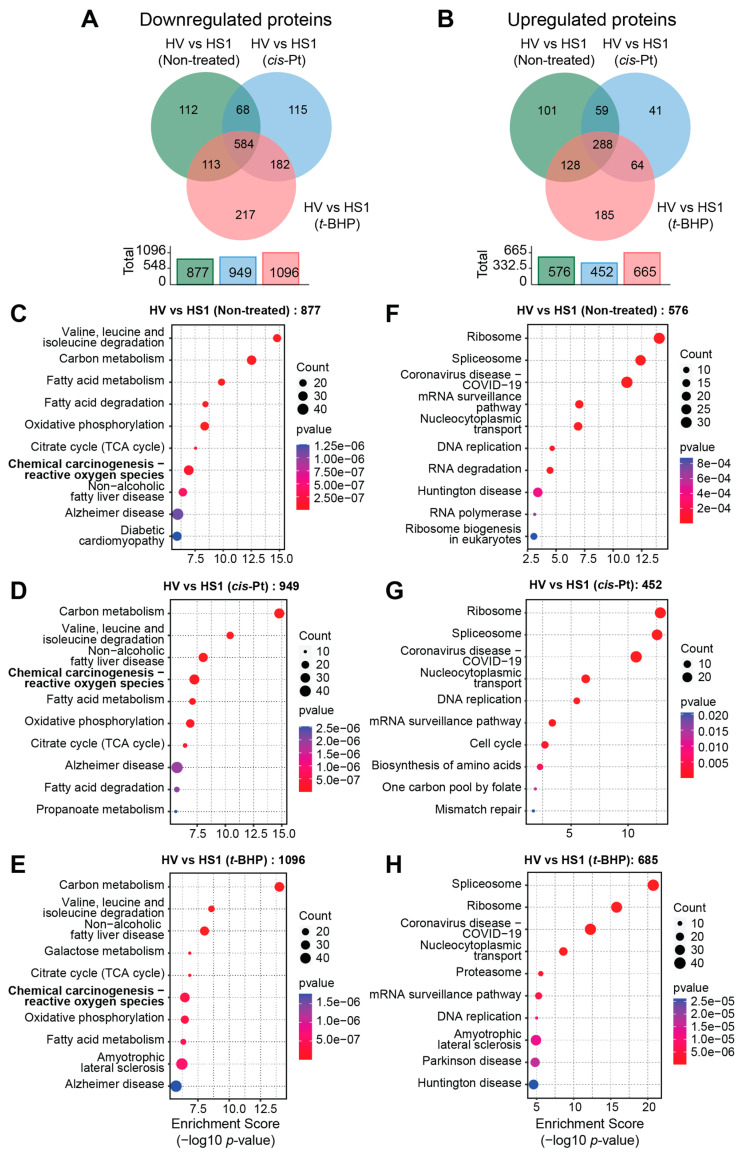
Pathway analysis of proteins downregulated or upregulated by SOCS1. (**A**,**B**) Venn diagrams showing the numbers of proteins downregulated (**A**) or upregulated (**B**) in untreated, cisplatin or *t*-BHP treated Hepa-SOCS1 cells compared to respective Hepa-Vector cells. Total number of downregulated and upregulated proteins are indicated in the histograms below the Venn diagrams. (**C**–**H**) Pathway analysis of all proteins downregulated or upregulated in untreated (**C**,**F**), cisplatin-treated (**D**,**G**) or *t*-BHP-treated (**E**,**H**) Hepa-SOCS1 cells.

Next, we compared the proteins modulated by SOCS1 in hepatoma cells after segregating them into downregulated or upregulated proteins. Proteins downregulated by SOCS1 were more numerous than upregulated proteins in untreated as well as in cisplatin- or *t*-BHP-treated cells (Figure 9A,B). Within the downregulated and upregulated proteins, more than half of the proteins were shared by all three groups (584 and 288, respectively; Figure 9A,B), reflecting the trend with total modulated proteins (Figure 8A). Similarly, a significant number of downregulated and upregulated proteins were shared between untreated and cisplatin- or *t*-BHP-treated cells, and cisplatin-treated and *t*-BHP-treated cells (Figure 9A,B). The pathway analysis of proteins significantly downregulated or upregulated by SOCS1 revealed that the ‘chemical carcinogenesis–reactive oxygen species’ and ‘carbon metabolism’ pathway proteins were downregulated (Figure 9C–E), whereas the ‘ribosome’ proteins were upregulated (Figure 9F–H) in untreated as well as in cisplatin- or *t*-BHP-treated cells. These results indicated that SOCS1 reduces the expression of proteins involved in oxidative stress response, thereby reducing the ability of Hepa-SOCS1 cells to tolerate oxidative stress.

**Figure 10:**
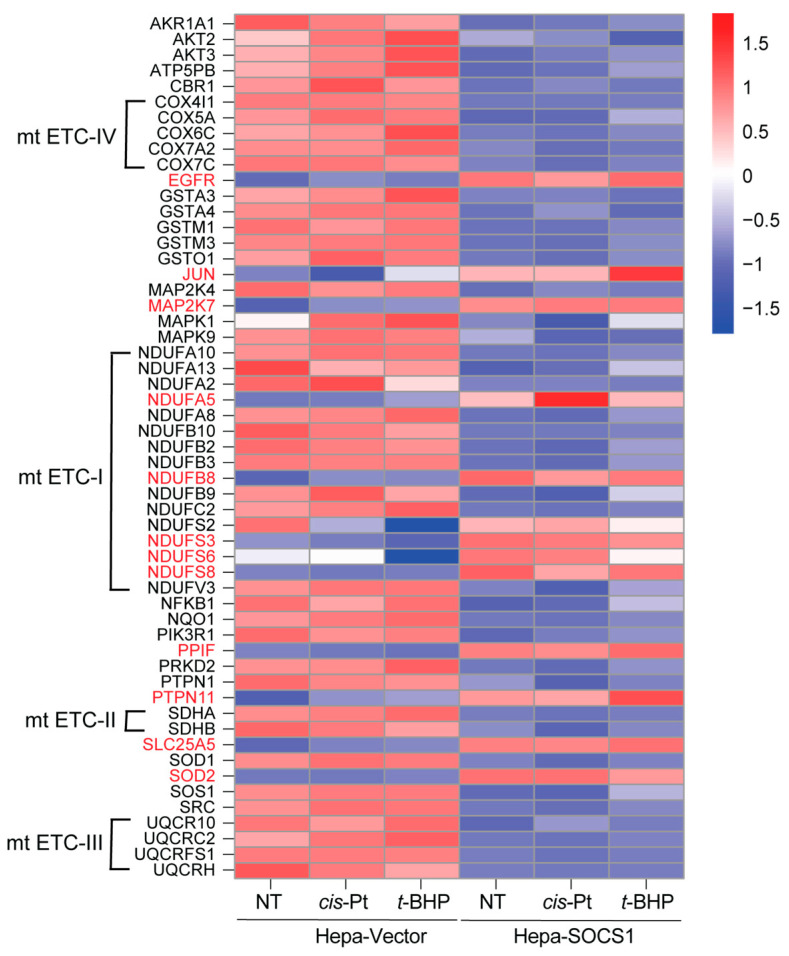
Heatmap analysis of the ‘chemical carcinogenesis–reactive oxygen species’ pathway proteins modulated by SOCS1. All proteins of the ‘chemical carcinogenesis–reactive oxygen species’ pathway modulated in untreated, cisplatin-treated and *t*-BHP-treated Hepa-SOCS1 cells compared to Hepa-Vector controls were pooled and their signal intensities are plotted in a heatmap. Proteins that are upregulated in Hepa-SOCS1 cells are identified in red colored font. Members of the mitochondrial ETC complexes are indicated.

Next, we pooled all proteins of the ‘chemical carcinogenesis–reactive oxygen species’ pathway found to be modulated in untreated, cisplatin-treated and *t*-BHP-treated Hepa-SOCS1 cells compared to Hepa-Vector controls and carried out a heatmap analysis (Figure 10). A large proportion of these proteins were downregulated in Hepa-SOCS1 cells compared to Hepa-Vector cells; however, many proteins showed an inverse pattern (e.g., SOD1 versus SOD2). Consistent with the data shown in Figure 6, there was only a modest difference in expression between untreated and treated conditions either in Hepa-Vector or in Hepa-SOCS1 cells, although certain notable changes following oxidative stress were also observed (e.g., JUN, NDUFA5, NDUFA8). Among the prominent NRF2 target proteins, GSTM1 and NQO1 were markedly downregulated in untreated and cisplatin- or *t*-BHP-treated Hepa-SOCS1 cells compared to Hepa-Vector controls. Notably, SDHA (succinate dehydrogenase complex subunit A), a tumor suppressor that allows cells to grow in low oxygen conditions, was markedly downregulated by SOCS1. A string analysis of these proteins indicated that they include 33 of the 104 unique proteins of the mitochondrial electron transport chain (mt ETC) complexes (Supplementary Figures S4A,B). Whereas many members of complex-I, II, III and IV were downregulated, certain members of complex-I were upregulated in Hepa-SOCS1 cells (Figure 10).

### 3.8. Modulation of Peroxiredoxins by SOCS1

**Figure 11:**
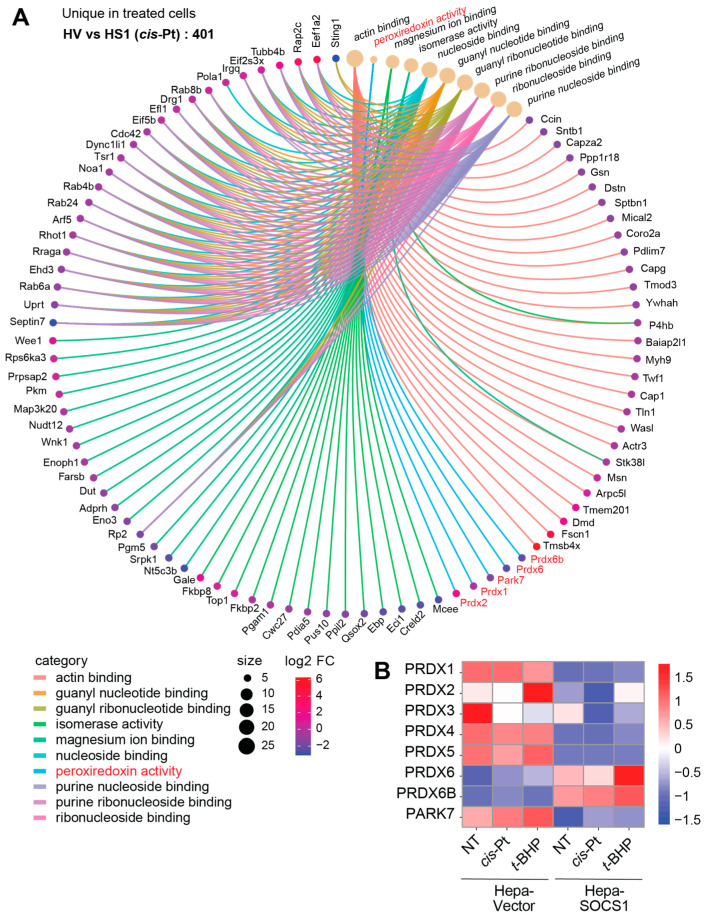
Modulation of peroxiredoxins by SOCS1. Proteins that were enriched in cisplatin-treated Hepa-SOCS1 cells compared to Hepa-Vector controls were analyzed by CNET plot (**A**). Peroxiredoxin pathway proteins are indicated in red color. (**B**) Signal intensities of the modulated peroxiredoxin family proteins in untreated, cisplatin-treated and t-BHP-treated Hepa-Vector and Hepa-SOCS1 cells arecompared by heatmap analysis.

Gene ontology analyses indicated that proteins involved in ribosome biogenesis, ribonuleoprotein assembly and mRNA processing predominate the total proteins modulated by SOCS1 in Hepa cells at steady state as well as among the proteins uniquely modulated in Hepa-SOCS1 cells after cisplatin or *t*-BHP treatment (Supplementary Figure S5). These results suggest an important role for SOCS1 in regulating cellular protein homeostasis through modulating mRNA processing and its translation by ribosomes. Notably, proteins modulated by SOCS1, uniquely in cisplatin-treated cells, showed a significant enrichment of ‘peroxiredoxin activity’ (Supplementary Figure S5), which is recognized as an important pathway in cellular redox homeostasis and is implicated in cancer development and progression [59,60,61]. The CNET plot of the proteins uniquely modulated by SOCS1 revealed that several peroxiredoxin family proteins are modulated by SOCS1 in cisplatin-treated Hepa-SOCS1 cells (Figure 11A). Heatmap analysis revealed that among the peroxiredoxin family proteins, PRDX2 is markedly upregulated in SOCS1-expressing cells, and PRDX1 and PRDX4 showed a moderate upregulation, whereas PRDX3, PRDX6 and PRDX6b were downregulated (Figure 11B). These observations suggest that the SOCS1-dependent regulation of cellular redox homeostasis is mediated not only by enzymes and proteins induced by NRF2, but also by peroxiredoxins.

## 4. Discussion

Whereas the tumor suppressor role of SOCS1 has been genetically confirmed in mouse models of colorectal cancer and hepatocellular carcinoma [30,31,32], the mechanisms of SOCS1-mediated tumor suppression are not yet completely understood. SOCS1 is implicated in regulating JAK-STAT and RTK signaling, inflammatory signaling pathways, p53 functions and the oncogenic potential of the oncojanus proteins p21 and NRF2 [13,15,31,32,33,34,35]. Our investigations on the role of SOCS1 in regulating NRF2-mediated oxidative stress response revealed that SOCS1 might also modulate ROS generation and other antioxidant response pathways, which can impact cancer progression and the efficacy of cancer therapies.

Reactive oxygen species emerged before life and became an essential feature of oxygenic life as living organisms evolved ways to use, generate and regulate ROS [62,63]. In mammalian cells, endogenous ROS are produced mainly as by-products of oxidative phosphorylation through the mitochondrial ETC that generates superoxide, which is converted to H_2_O_2_ by superoxide dismutase (SOD2) and transported to the cytosol [64]. NADPH oxidases (NOX) on the plasma membrane also produce H_2_O_2_ following activation by diverse stimuli including pathogens, cytokines, growth factors, signaling lipids and mechanical stimuli [65]. H_2_O_2_ is a key mediator of signal propagation via the thiol oxidation of many signaling molecules (redox signaling) in response to extracellular cues. Redox signaling is exploited by cancer cells, which produce higher levels of endogenous ROS, to their own advantage [66,67,68]. However, the accumulation of ROS and their excess production are detrimental, causing apoptosis, ferroptosis and necroptosis [64,69]. Hence, cells have devised multiple ways to inactivate ROS, with NRF2 and p53 playing key roles in maintaining the redox balance [70,71,72,73]. The three major antioxidant enzymatic mechanisms that inactivate H_2_O_2_ include peroxiredoxins (PRXs), glutathione peroxidases (GPXs) and catalase [64,68,74]. Many protein components of cellular redox regulation machinery are regulated by NRF2 when oxidative stress dislodges KEAP, allowing the transcriptional activation of NRF2 [68,71]. In addition to KEAP1 oxidation, NRF2 is also activated by other mechanisms, including p21 [38,70]. Cancer cells, which display a higher metabolic rate and consequently experience more ROS production, upregulate NRF2 to counter the elevated oxidative stress while exploiting ROS for cancer cell signaling and disease progression [36,64,75]. ROS is also produced by noxious exogenous stimuli such as radiation and heavy metals, and the induction of cell death via increasing oxidative stress to unsustainable levels is one of the mechanisms underlying anticancer agents such as anthracyclins and cisplatin [64,75,76]. Our findings, that stable SOCS1 expression in HCC cells downregulates NRF2 expression and sensitizes them to cisplatin-mediated death, indicate that the loss of functional SOCS1 in many cancers [18,19,20,21,22,23,24,25,26,27,28] would allow them to withstand higher levels of oxidative stress, promote cancer progression and even promote resistance to cancer therapies that induce ROS-mediated cell death.

Even though cisplatin causes nuclear DNA damage, its antitumor cytotoxic effects are also mediated via the induction of mitochondrial ROS and possibly via NOX activation as well [45,46,47]. Consistent with the finding that SOCS1 attenuates the induction of NRF2-dependent antioxidant response genes, Hepa-SOCS1 cells showed increased sensitivity to cisplatin-induced cell death. Moreover, the proteomic analysis revealed a marked downregulation of many proteins within complex-I (NADH:ubiquinone oxidoreductase), complex-II (succinate:Ubiquinone oxidoreductase), complex-III (ubiquinol-Cytochrome C reductase) and complex-IV (Cytochrome oxidase) of the mitochondrial ETC, as well as a marked reduction in the cytosolic/mitochondrial inner membrane SOD (SOD1), although there was a modest increase in mitochondrial SOD (SOD2) in Hepa-SOCS1 cells at steady state and after cisplatin treatment (Figure 10). SOD1 is constitutively expressed, whereas SOD2 is induced by oxidative stress [77]. Hence, the upregulation of SOD2 in Hepa-SOCS1 could represent a compensatory mechanism of reduced SOD1 expression. As SOD1 and SOD2 are not regulated by NRF2 [77], how SOCS1 reduces the expression of SOD1 remains to determined. Nonetheless, our findings indicate that even though SOCS1 could impact mitochondrial ETC, and consequently mitochondrial ROS generation, these modulations do not seem to be detrimental to cell survival, as reduced cell viability was observed only after cisplatin treatment or exposure to the oxidizing agent *t*-BHP. As constitutive SOCS1 expression is negligible, but is rapidly upregulated following growth stimuli by cytokines, growth factors and oncogenes, it is likely that the induced SOCS1 might fine-tune mitochondrial ROS generation that would permit signal amplification while preventing a detrimental increase in oxidative stress. The regulation of mitochondrial ROS generation could be one of the reasons why SOCS1 expression is widely repressed in diverse cancers; however, further studies employing diverse growth stimuli are needed to test this hypothesis.

Another observation in our study is the modulation of peroxiredoxins in Hepa-SOCS1 cells. Peroxiredoxins (PRDXs), discovered many years after catalase and GPXs and originally considered to be much less efficient in neutralizing H_2_O_2_, are more abundant, distributed in different ROS-producing organelles and could actually account for more than 90% of peroxidase activity in the cytosol and mitochondria [59,74]. All six PRDXs (PRDX1-6) are implicated in tumorigenesis and disease progression in diverse cancers, although antitumor functions have also been reported [59,60]. Most of the PRDX proteins were downregulated in Hepa-SOCS1 cells at steady state and were not induced after oxidative stress (Figure 11B). Among the PRDX proteins, PRDX1 and PRDX6 are known NRF2 target genes [68,71,78], of which PRDX1 was downregulated but PRDX6 and PRDX6B upregulated in Hepa-SOCS1 cells. It is likely that the varied modulation of PRDX proteins by SOCS1 could be related to both redox regulation and tumor suppressor pathways.

An earlier report showed that SOCS1 upregulated thioredoxin (TXN), which regulates thiol-related signaling and serves as the ROS scavenging intermediate for PRDXs, as GSH is for GPXs [78,79], and was shown to be upregulated in SOCS1-expressing T lymphocytes treated with H_2_O_2_ and confers protection from apoptosis [80]. TRX and related proteins were not represented in the top-ranking signaling pathways modulated by SOCS1 in Hepa cells. However, the analysis of the expression of TXN and related proteins revealed that TXN is highly expressed in Hepa-Vector cells, and was downregulated in Hepa-SOCS1 cells at steady state (Supplementary Figure S6). Even though TXN is an NRF2 target gene [71], it was upregulated by cisplatin or *t*-BHP in Hepa-SOCS1 cells, unlike several NRF2 target genes and proteins, which is partly in agreement with the H_2_O_2_-induced TXN upregulation in lymphocytes [80]. It is noteworthy that even though there is a consensus on core NRF2 target genes, there exists vast diversity in genes reported to be modulated by NRF2 in different experiments systems [81,82,83,84]. Strikingly, TXN interacting protein (TXNIP), a multifunctional protein that translocates to mitochondria to regulate TXN activity during oxidative stress and to promote apoptosis, was upregulated in Hepa-SOCS1 cells at steady state and after oxidative stress. Whether elevated TXNIP expression in Hepa-SOCS1 cells contributes, at least partly, to their reduced survival following exposure to cisplatin or *t*-BHP remains to be tested. Hepa-SOCS1 cells also showed a marked upregulation of a thioredoxin domain containing protein TXNDC12 at steady state that remained unchanged after oxidative stress. TXNDC proteins can exert antioxidant functions, and their elevated expression is associated with poor prognosis in many cancers [85,86]. A recent report implicated TXNDC12 in inhibiting lipid peroxidation and ferroptosis [87]. Even though SOCS1 has previously been shown to promote ferroptosis [88], it is possible that TXNDC12 was upregulated in Hepa-SOCS1 as a compensatory mechanism to maintain redox balance but contributed to the reduced lipid peroxidation in Hepa-SOCS1 cells following cisplatin or *t*-BHP, despite showing increased oxidative stress.

The tumor suppressor p53 orchestrates a range of antitumor mechanisms in response to oxidative stress. Depending on the severity of DNA damage, p53 can either facilitate DNA damage repair, induce senescence and cell cycle arrest, or promote apoptosis [89]. Given that SOCS1 can promote p53 activation [34,35], and considering that p21 is a key downstream effector of p53, it can be hypothesized that constitutive SOCS1 expression would amplify ROS-induced p53 activation. This, in turn, would elevate p21 expression, enhance NRF2 activation, promote the efficient neutralization of ROS and support cell survival. However, we have reported that SOCS1 directly interacts with p21, promotes its degradation, diminishes NRF2 induction and increases oxidative stress [31,32]. Hence, we postulate that the SOCS1-mediated degradation of p21 would counterbalance any increase in p21 levels arising from SOCS1-dependent p53 activation, ultimately leading to diminished NRF2 activation and increased cell death in SOCS1-expressing cancer cells exposed to oxidative stress.

Both SOCS1 and p53 are also implicated in ferroptosis, a regulated cell death process caused by iron-dependent lipid peroxidation [90]. SOCS1 promotes ferroptosis by reducing the expression of SLC7A11, a subunit of the glutamate/cystine antiporter that allows cystine influx needed for glutathione biosynthesis [88]. Notably, SLC7A11 is induced by NFR2 during oncogenic stress, and this induction is inhibited by p53 [91]. Hence, it is likely that the SOCS1-mediated inhibition of SLC7A11 also occurs through the inhibition of p21-mediated NRF2 activation rather than via p53 activation. While this hypothesis requires experimental validation, reduced lipid peroxidation in Hepa-SOCS1 cells (Figure 4) suggests that the SOCS1-mediated inhibition of SLC7A11 might only play a limited role in the heightened susceptibility to oxidative stress-induced cell death. However, the possibility that this mechanism might occur earlier in Hepa-SOCS1 cells compared to Hepa-Vector cells, thereby contributing to reduced lipid peroxidation in the former, should not be discounted.

A limitation of our study is the short duration of oxidative stress used for proteomic analyses. We chose the shorter oxidative stress time point to collect the proteome before the beginning of cell death, and to identify proteins that are modulated by SOCS1 under oxidative stress, as SOCS1 is known to promote ubiquitination and the proteasomal degradation of diverse proteins [15,16,17]. Even though a longer period of oxidative stress would have been informative, the current study clearly shows that SOCS1 expression in HCC cells increases their sensitivity to oxidative stress and that proteins involved in both ROS generation and detoxification are modulated by SOCS1.

Elevated oxidative stress, antioxidant mechanisms and oncogenic redox signaling are integral to cancer progression, aggressivity and resistance to chemotherapy [67,76,92]. Hence, the modification of ROS levels in cancer cells is an active area of cancer therapeutics. Whereas suppressing ROS production or antioxidant treatment would cause cytostasis in cancer cells, increasing ROS production or suppressing antioxidant systems would elevate ROS to unsustainable levels, leading to oxidative cell death. These approaches would not impact normal cells, as their highly regulated ROS production and antioxidant response systems would maintain homeostasis. In this context, SOCS1-deficient HCC cells, which upregulate NRF2-mediated and possibly other antioxidant defense mechanisms, could be rendered susceptible to chemotherapeutic agents that increase ROS production if their elevated antioxidant mechanisms could be simultaneously suppressed. However, the targeting of NRF2 without compromising its homeostatic functions in normal cells would be a daunting task [93]. Nonetheless, other approaches targeting cells that express elevated levels of NRF2 targets such as NQO1 [94] offer hope to induce oxidative cell death in cancer cells with exaggerated antioxidant defenses.

## 5. Conclusions

This study provides insight into the tumor suppression mechanisms of SOCS1. SOCS1 reduces NRF2 induction and NRF2-driven antioxidant defenses. SOCS1 also modulates the expression of proteins involved in mitochondrial ROS generation, as well as other pathways of ROS inactivation including peroxiredoxin and thioredoxin systems. The combined effects of SOCS1-mediated redox regulation likely lower the threshold level of ROS tolerance in cancer cells with intact SOCS1. These regulatory mechanisms will be lost in cancers with *SOCS1* gene repression, raising the threshold level of ROS tolerance and thereby diminishing the cytotoxic effects of endogenous and drug-induced ROS. In such SOCS1-deficient tumors, drugs that lower antioxidant defenses would be helpful in increasing sensitivity to drugs that mediate tumor killing by increasing ROS production.

## Data Availability

The mass spectrometry data are deposited to the ProteomeXchange Consortium via the PRIDE [95] partner repository with the dataset identifier PXD047908. Curated datasets and comparisons between groups are provided in Supplementary Table S3.

## Supplementary Materials

The following supporting information can be downloaded at: https://www.mdpi.com/article/10.3390/cancers16020292/s1, Figure S1: SOCS1 diminishes NRF2 induction following oxidative stress in human Hep3B cells; Figure S2: SOCS1 does not impact SOCS3 protein expression induced by oxidative stress; Figure S3: Correlation between the expression of NRF2 target genes and *NFE2L2* and *SOCS1* expression in the TCGA-LIHC transcriptomic data; Figure S4A: STRING analysis of proteins modulated by SOCS1 and represented in the ‘chemical carcinogenesis–reactive oxygen species’ pathway; Figure S4B: Proteins modulated by SOCS1 that are involved in the electron transport chain of the mitochondrial oxidative phosphorylation; Figure S5: Gene ontology analysis of all proteins modulated by SOCS1; Figure S6: Expression of thioredoxin and related proteins in untreated, cisplatin-treated and *t*-BHP treated Hepa-Vector and Hepa-SOCS1 cells; Table S1: List of antibodies used in this study; Table S2: List of RT-qPCR primers used in this study; Table S3: Curated datasets and comparisons between groups.
