# Supplementary material for: The Tumor Suppressor SOCS1 Diminishes Tolerance to Oxidative Stress in Hepatocellular Carcinoma
Source: Cancers (Basel). 2024 Jan 10;16(2):292. doi: 10.3390/cancers16020292 (PMC10814246; doi:10.3390/cancers16020292)

The tumor suppressor SOCS1 diminishes tolerance to oxidative stress in hepatocellular carcinoma

Akhil Shukla et al.,

Supplementary Figures S1 to S6

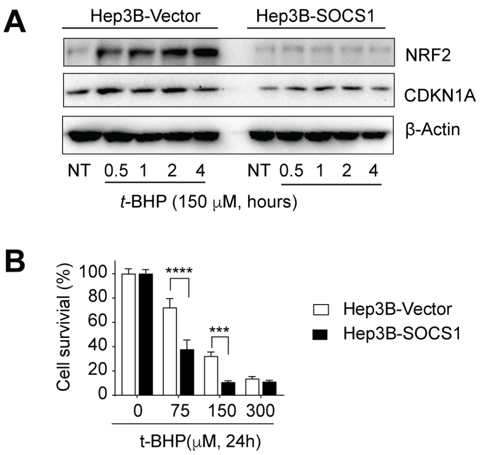

Supplementary Figure S1. SOCS1 diminishes NRF2 induction following oxidative stress in human Hep3B cells. (A) Hep3B-vector and Hep3B-SOCS1 cells were treated with *t*-BHP (150 μM) and evaluated for the expression of p21 and NRF2 proteins by western blot. Representative data from two independent experiments are shown. (B) Hep3B-vector and Hep3B-SOCS1 cells were treated with *t*-BHP (150 μM). Cell viability was assessed after 24h. Cumulative data from three separate experiments are shown as Mean ± SE. ANOVA with Tukey's multiple comparison test. *p* values: * <0.05, ** <0.01, *** <0.001, **** <0.001.

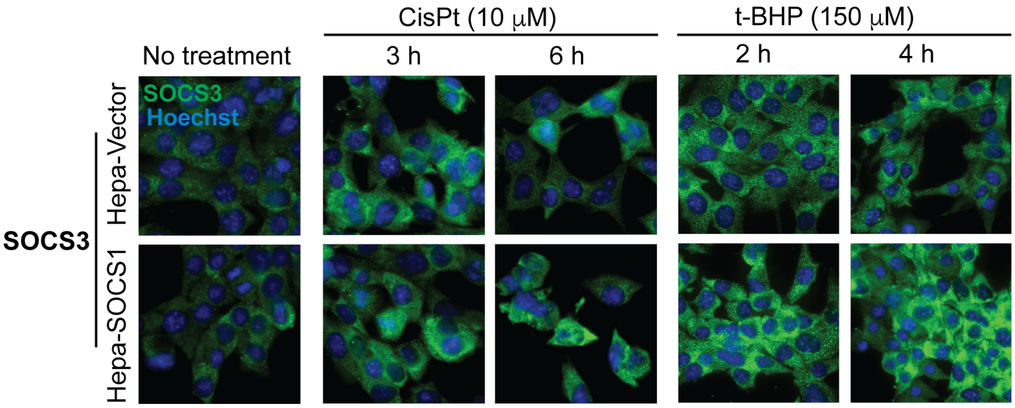

Supplementary Figure S2. SOCS1 does not impact SOCS3 protein expression induced by oxidative stress. Hepa-Vector and Hepa-SOCS1 cells grown on glass cover slips were treated with cisplatin (10 μM) or *t*-BHP (150 μM) for the indicated periods of time. Expression of the SOCS3 protein was evaluated by immunofluorescence microscopy. Representative data from two experiments are shown.

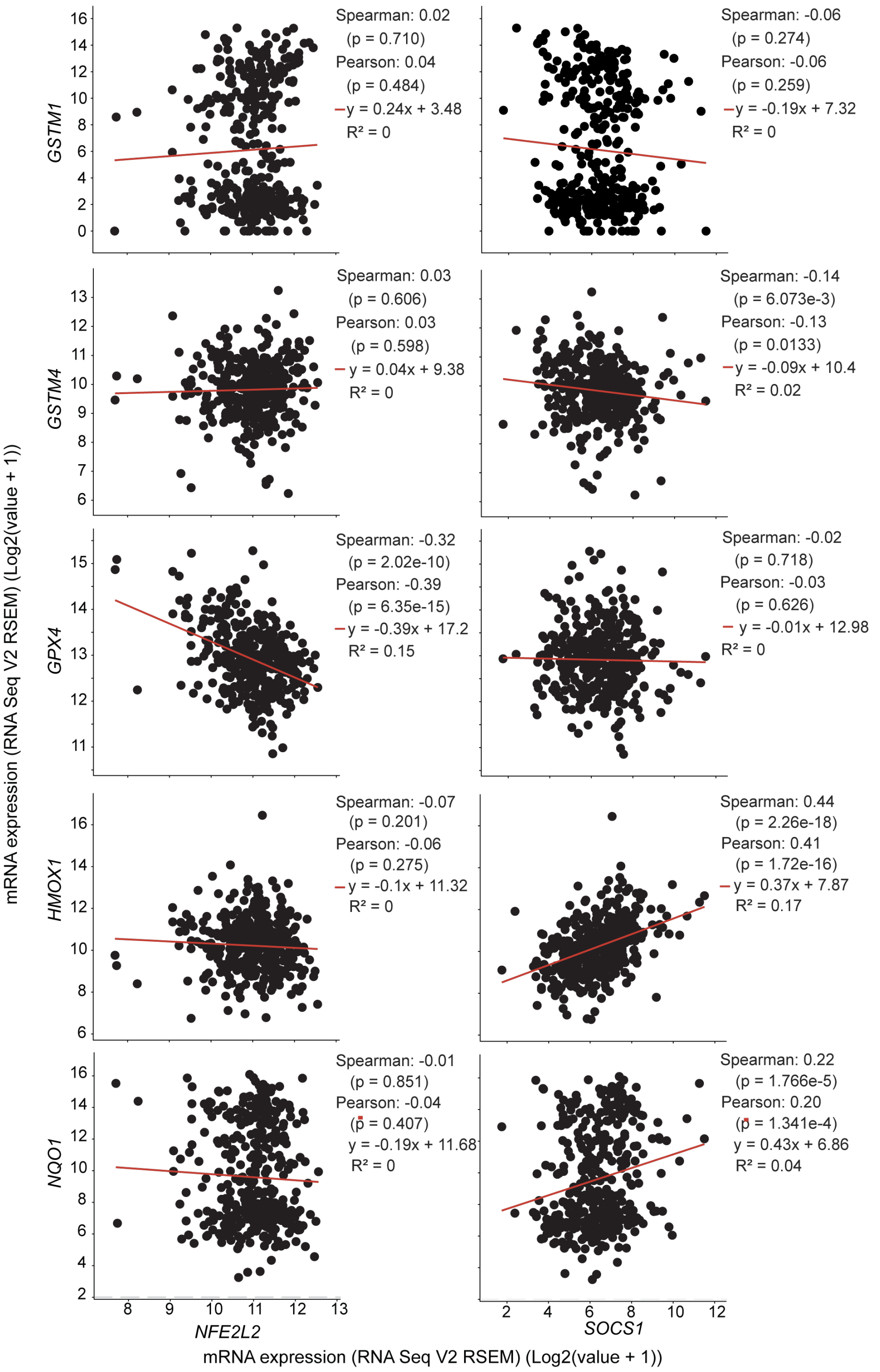

Supplementary Figure S3. Correlation between the expression of NRF2 target genes and *NFE2L2* and *SOCS1* expression in the TCGA-LIHC transcriptomic data.

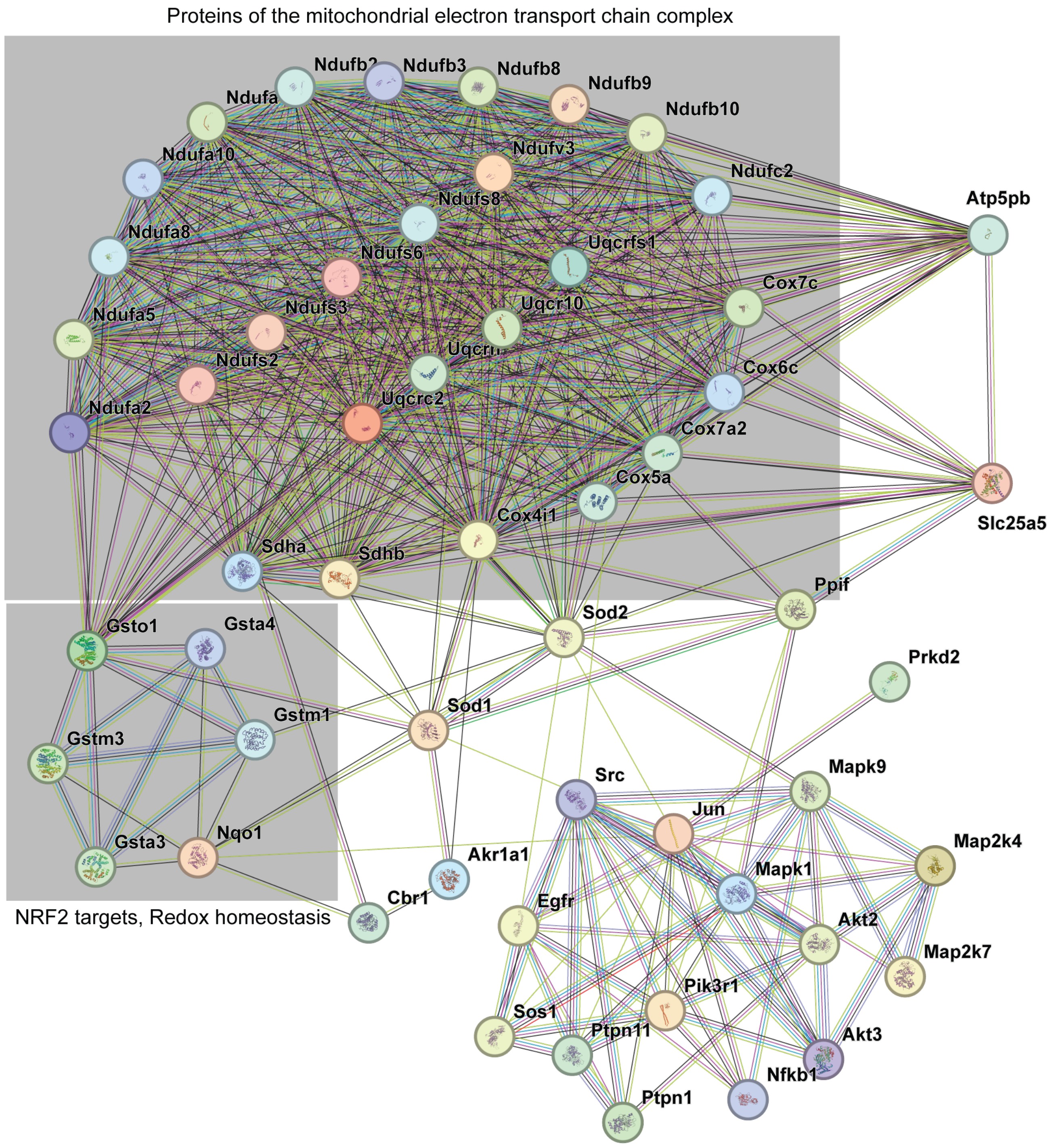

Supplementary Figure S4A. STRING analysis of proteins modulated by SOCS1 in the ‘chemical carcinogenesis- reactive oxygen species’ pathway. All proteins of the ‘chemical carcinogenesis- reactive oxygen species’ pathway modulated in untreated, cisplatin-treated and *t*-BHP-treated Hepa-SOCS1 cells compared to Hepa-Vector controls were analysed for protein interaction network using the STRING database (https://string-db.org; accessed in November 2023). Proteins that form the mitochondrial electron transport chain and the NRF2-induced proteins and those implicated in Redox homeostasis are indicated within grey boxes.

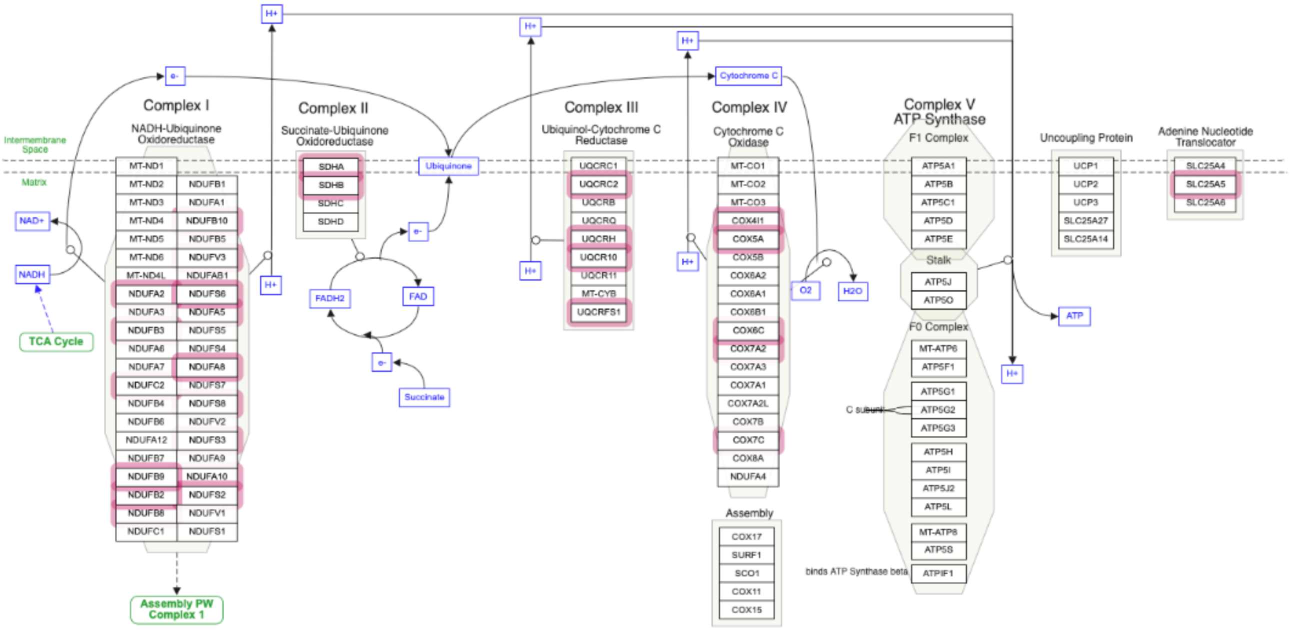

Supplementary Figure S4B. Proteins modulated by SOCS1 shown in Figure 4A were analyzed using the NDEx Integrated Query tool (https://www.ndexbio.org; Accessed on Dec 2023; v2.5.5), which identified their enrichment within the Wikipathway WP111- Electron transport OXPHOS system in mitochondria – *Homo Sapiens*; p-value: 4.97e -38). Identified proteins are highlighted with pink boundaries.

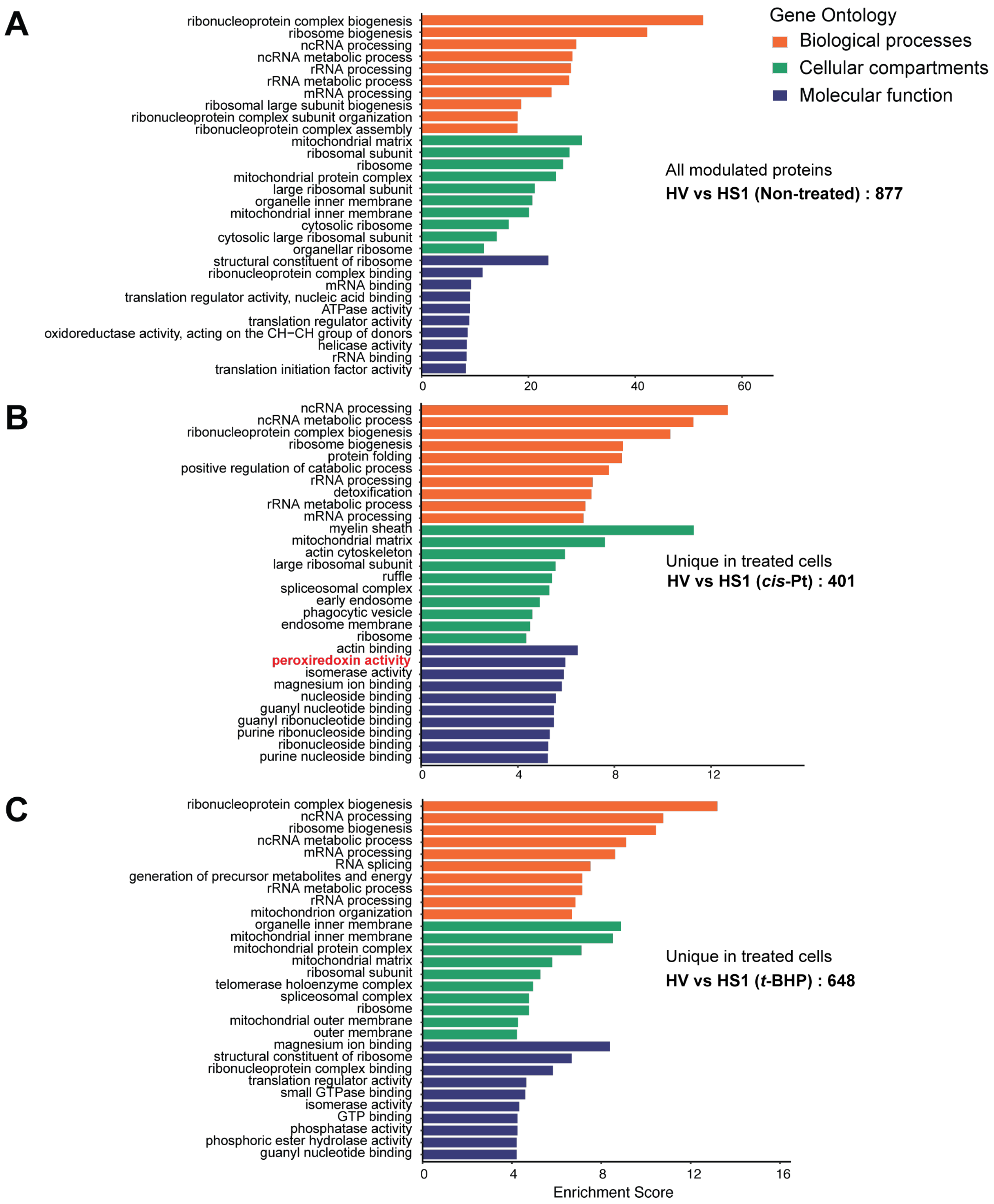

Supplementary Figure S5. Gene ontology analysis of all proteins modulated by SOCS1 in untreated Hepa-SOCS1 cells (A), and proteins uniquely modulated in cisplatin-treated (B) and *t*-BHP treated (C) compared to Hepa-Vector controls. Total number of proteins included in these analyses are indicated.

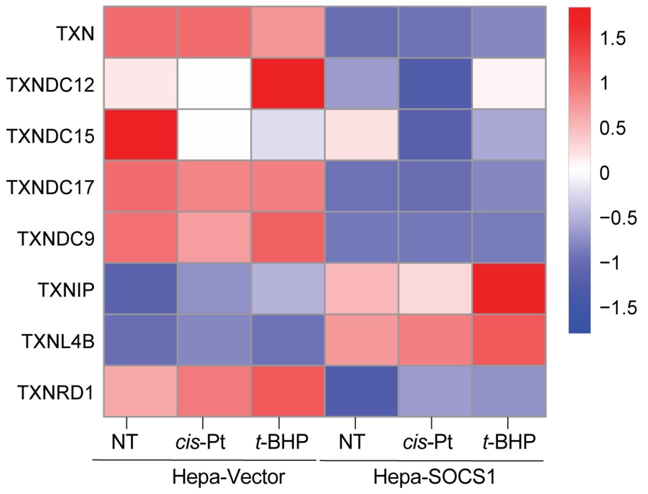

Supplementary Figure S6. Expression of thioredoxin and related proteins in untreated, cisplatin-treated and *t*-BHP treated Hepa-Vector and Hepa-SOCS1 cells. TXNDC12, thioredoxin domain contain protein 12; TXNIP, thioredoxin interacting protein; TXNL, thioredoxin-like.

Uncropped western blots: Figure S1A

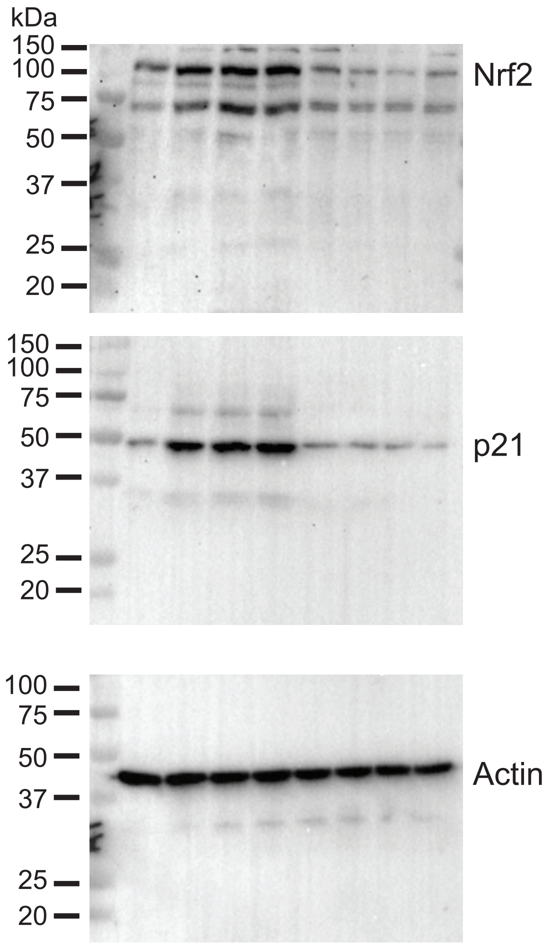

Uncropped western blots: FigureS1B

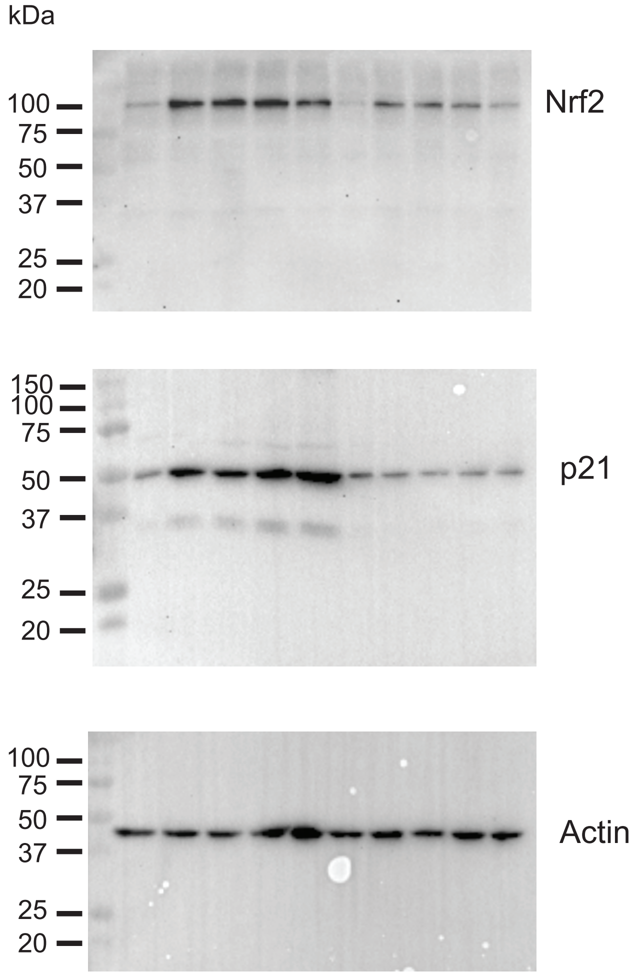

Uncropped western blots: Figure S1A

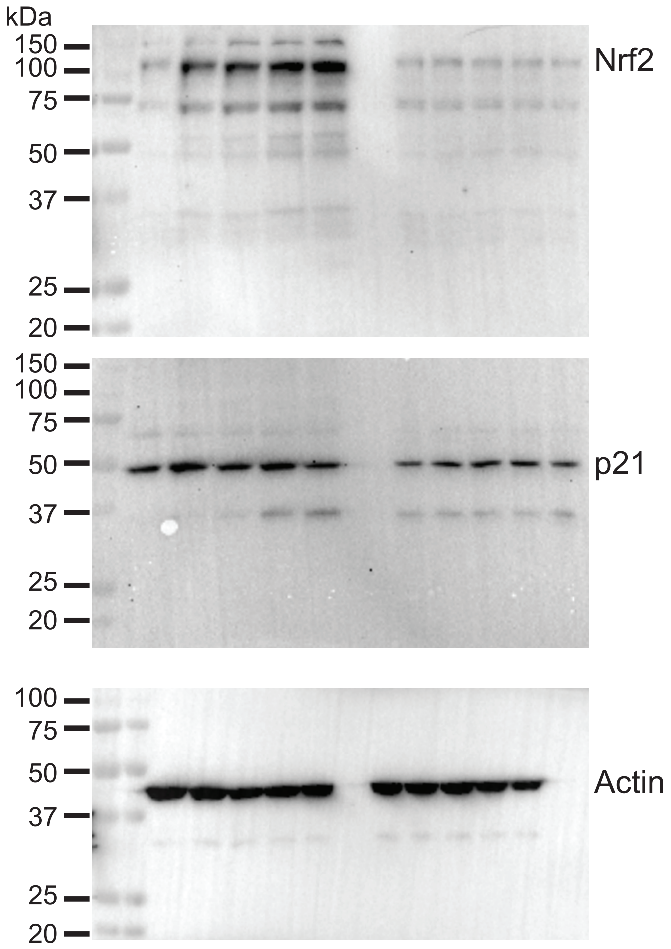

Supplement: Supplementary file 1 [file cancers-16-00292-s001.zip › cancers-2808381-supplementary.pdf]
